# Differences of gut microbiota and behavioral symptoms between two subgroups of autistic children based on γδT cells-derived IFN-γ Levels: A preliminary study

**DOI:** 10.3389/fimmu.2023.1100816

**Published:** 2023-02-15

**Authors:** Xin-Jie Xu, Ji-Dong Lang, Jun Yang, Bo Long, Xu-Dong Liu, Xiao-Feng Zeng, Geng Tian, Xin You

**Affiliations:** ^1^ Medical Science Research Center, Research Center for Translational Medicine, Department of Scientific Research, Peking Union Medical College Hospital, Beijing, China; ^2^ Department of Rheumatology and Clinical Immunology, Peking Union Medical College Hospital, Chinese Academy of Medical Sciences and Peking Union Medical College, Beijing, China; ^3^ Precision Medicine Center, Geneis Beijing Co., Ltd., Beijing, China; ^4^ Key Laboratory of Rheumatology and Clinical Immunology, Ministry of Education, National Clinical Research Center for Dermatologic and Immunologic Diseases (NCRC-DID), Beijing, China; ^5^ Autism Special Fund, Peking Union Medical Foundation, Beijing, China

**Keywords:** autism spectrum disorders, gut microbiota, metagenomics, interferon, immune

## Abstract

**Background:**

Autism Spectrum Disorders (ASD) are defined as a group of pervasive neurodevelopmental disorders, and the heterogeneity in the symptomology and etiology of ASD has long been recognized. Altered immune function and gut microbiota have been found in ASD populations. Immune dysfunction has been hypothesized to involve in the pathophysiology of a subtype of ASD.

**Methods:**

A cohort of 105 ASD children were recruited and grouped based on IFN-γ levels derived from *ex vivo* stimulated γδT cells. Fecal samples were collected and analyzed with a metagenomic approach. Comparison of autistic symptoms and gut microbiota composition was made between subgroups. Enriched KEGG orthologues markers and pathogen-host interactions based on metagenome were also analyzed to reveal the differences in functional features.

**Results:**

The autistic behavioral symptoms were more severe for children in the IFN-γ-high group, especially in the body and object use, social and self-help, and expressive language performance domains. LEfSe analysis of gut microbiota revealed an overrepresentation of *Selenomonadales*, *Negatiyicutes*, *Veillonellaceae* and *Verrucomicrobiaceae* and underrepresentation of *Bacteroides xylanisolvens* and *Bifidobacterium longum* in children with higher IFN-γ level. Decreased metabolism function of carbohydrate, amino acid and lipid in gut microbiota were found in the IFN-γ-high group. Additional functional profiles analyses revealed significant differences in the abundances of genes encoding carbohydrate-active enzymes between the two groups. And enriched phenotypes related to infection and gastroenteritis and underrepresentation of one gut–brain module associated with histamine degradation were also found in the IFN-γ-High group. Results of multivariate analyses revealed relatively good separation between the two groups.

**Conclusions:**

Levels of IFN-γ derived from γδT cell could serve as one of the potential candidate biomarkers to subtype ASD individuals to reduce the heterogeneity associated with ASD and produce subgroups which are more likely to share a more similar phenotype and etiology. A better understanding of the associations among immune function, gut microbiota composition and metabolism abnormalities in ASD would facilitate the development of individualized biomedical treatment for this complex neurodevelopmental disorder.

## Introduction

Autism spectrum disorders (ASDs) are defined as a group of pervasive neurodevelopmental disorders characterized by persistent deficits in social communication and social interaction, plus restricted, repetitive patterns of behavior, interests, or activities (1). The exact etiology of ASD is still unknown and accumulated results of recent studies suggest that instead of a single causative factor, ASD may be caused by the combined effects and interplay between genetic heritability and complex environmental risk factors (2–5).

The heterogeneity in the symptomology and etiology of ASD has long been recognized by clinicians and researchers (6–12). A deep insight into this heterogeneity and using appropriate strategy to identify ASD subtypes are crucial, since different subtypes may result from different pathophysiology and respond differently to certain therapies (6, 9, 11–13). Previous subtyping methods mainly used behavioral characteristics and intellectual functioning as indicators (6, 10, 14–16). In recent years, subtyping ASD individuals according to their co-occurring medical disorders or associated physiological abnormalities has also emerged and got accumulated promising results (12, 17–20).

It has been demonstrated that certain immune-mediated conditions (such as allergies and some autoimmune diseases) were more prevalent in ASD subjects (21–24). Immune dysfunction has been hypothesized to involve in the pathophysiology of a subtype of ASD (12, 20, 25). Compared to behavioral symptoms, immune abnormalities are more objective since they can be measured using clinical and laboratory characteristic, thus would be a potential ideal subtyping indicator (25). Gamma delta (γδ) T cells play important roles in inflammatory and autoimmune diseases (26). They add to the imbalanced pro- and anti-inflammatory reactions and recruit other immune cells such as macrophages (27). IFN-γ is one of the major cytokines produced by γδ T cells. Results from several previous studies revealed elevated IFN-γ levels in different tissues in some but not all ASD subjects (28–31), indicating that IFN-γ might participate in the immune dysfunction associated with certain subtype of ASD.

The crosstalk between gut microbiota and host immune function has been increasingly recognized in recent years (32–35). Gut microbiota can interact with immune cells and modulate the function of immune system, and inflammation, which is caused by abnormal immune responses, can also influence the composition of the gut microbiome (32, 34). Moreover, differences in the intestinal microbial community have also been found in children with ASD as compared to neurotypicals (17, 36–39). Since gut microbes can communicate with the host brain through multiple ways, including neuroactive compounds, toxin metabolites and immune modulation, it is suggested that alterations of gut-immune-brain axis play critical roles in ASD (38, 40–43). A better understanding of the association and comprehensive interactions among gut microbiota composition, immune characterization and behavioral symptoms in ASD, and subtyping this heterogenous disorder based on objective immunological characteristics into more homogeneous subgroups will not only provide useful information on the biological mechanisms underlying the pathogenesis of ASD, but also facilitate the development of individualized therapy strategies for ASD population (9, 18, 20).

In the present study, we recruited a cohort of 105 ASD children, and levels of IFN-γ derived from γδ T cells were measured to cluster children into subgroups. Comparison of autistic symptoms and gut microbiota composition was made between subgroups. Enriched KEGG orthologues markers and pathogen-host interactions based on metagenome were also analyzed to reveal the differences in functional features. Results of this study will provide additional evidence to support the association of gut microbiota alterations and immune dysfunction in ASD and suggest that IFN-γ could serve as a potential candidate biomarker to subtype ASD individuals into subgroups which tend to share a more similar phenotype and etiology.

## Materials and methods

### Participants

The study was approved by the Institutional Review Board of Peking Union Medical College Hospital (IRB #ZS-824). Autistic children were consecutively recruited from the Herun Clinic in Beijing, China. The inclusion criteria for autistic children were (1): Being diagnosed with autism which was confirmed by experienced psychiatrists according to the Diagnostic and Statistical Manual of Mental Disorders Fifth Edition (DSM-V, 2013) criteria (2). Free of antibiotic treatment, prebiotics and probiotics for at least 4 weeks before sample collection (3). The children’s primary caregivers had good reading and comprehension skills and were able to fill in the relevant assessment scales (4). The children’s parents or legal guardians volunteered to participate in this study and signed the informed consent. Autistic children with symptoms of other comorbid neurological or psychiatric disorders as confirmed by experienced clinicians or psychiatrists were excluded from the study. Detailed information on the purposes and procedures of the study were explained to the children’s parents or legal guardians. Written forms of full informed consent were obtained before involving the children in the study.

### Assessment of autistic symptoms

The following scales were used to assess autistic symptoms in children:

1. Autism Behavior Checklist (ABC). A behavior checklist consists of 57 items in 5 categories: sensory, relating, body and object use, language, and social and self-help (44). The scale utilizes an observer’s rating of the child’s behavior to quantify behaviors typically associated with autism. The children’s parents or primary caregivers were asked to fill out the ABC questionnaire to preliminarily evaluate the severity of ASD.
2. Autism Treatment Evaluation Checklist (ATEC). A checklist designed to be completed by parents, teachers, or caretakers, which could be used to monitor the general well-being of an individual over time. It consists of 4 subscales: speech/language communication, sociability, sensory/cognitive awareness, and health/physical/behavior. The validity of ATEC has been confirmed in several studies, and lower scores indicated fewer problems (45).
3. Clinical Language Status Questionnaire (CLSQ). A clinical language assessment questionnaire which was developed by Frank H Duffy et al. CLSQ could be used to evaluate the child’s current best expressive and receptive language performance with good reliability, and higher scores indicated better performances (46).

### Detection of IFN-γ expression in γδ T cells

Two milliliters of fasting venous blood samples were collected into chilled heparin tubes by trained nurses between 8:00 and 10:30 a.m. The samples were then diluted with equal volume of PBS, and the peripheral blood mononuclear cells (PBMCs) were separated by Ficoll (Tianjin Hao Yang Biological Technology Company) centrifugation.

Isolated PBMCs were cultured in 24 well plates with complete medium (RPMI 1640 medium (Hyclone) with fetal bovine serum (10%, Gibco), penicillin and streptomycin (100 u/ml)) and stimulated with 50ng/ml phorbol 12-myristate 13-acetate (PMA) (50ng/ml, Sigma) and 1µg/ml lonomycin (1ug/ml, Sigma) overnight. Brefeldin A (BFA) (Golgiplug, BD) was added in one hour after adding PMA and Ionomycin to block the secretion of the cytokines.

The stimulated PBMCs were washed twice with PBS, centrifuged and then resuspended. Subsequently, CD3-PE conjugated antibody (BD Pharmingen), TCRγδ-FITC conjugated antibody (Biolegend) was added to the cells. After 30 minutes of incubation at 4°C avoiding light, the cells were washed twice with PBS. The cells were then permeabilized for staining of intercellular cytokine with Cytoperm/Cytofix Fixation/Permeabilization Kit (BD). Subsequently, cells were incubated with APC-conjugated IFN-γ antibody (BD Pharmingen) for an hour. Then the cells were washed and resuspended with PBS, followed by flow cytometry assessment. Flow cytometry was performed on BD Accuri C6 flow cytometer, and the following data analysis was conducted with CFlow Plus 1.0.164.15.

### Fecal sample collection and DNA extraction

Children’s fresh fecal samples were obtained at home or Herun Clinic, immediately transferred into 1.5 ml sterile Eppendorf tubes (Axygen), and frozen into dry ice. All samples were stored at -80°C until analysis. DNA was extracted from fecal samples using the MO-BIO PowerSoil DNA Isolation Mini-Kit (Carlsbad) according to the protocol. DNA quality was assessed and controlled using gel electrophoresis.

### Metagenomic library construction and sequencing

The sequencing library construction and template preparation was performed using the NEBNext UltraTM DNA Library Prep Kit (New England Biolabs) following manufacturer’s instructions (input DNA >100 ng). Each sample was barcoded and equal quantities of barcoded libraries were used for sequencing. The quality and quantity of the libraries were assessed using the Agilent 2100 High Sensitivity DNA Kit (Agilent Technologies) and the ABI 7500 Real Time PCR System (Applied Biosystems) before Illumina sequencing. Illumina HiSeq 2500 and Hiseq X Ten sequencing systems were used for paired-end 150bp sequencing. Data with adaptor contamination and low-quality reads were discarded from the raw data. We acquired ~223Gb high-quality data for the 38 samples with an average of ~5.9Gb per sample.

### Data analysis

Taxonomic assignment of the main bacteria and the relative species abundances were calculated using MetaPhlAn (version 1.7.7) (47). Biodiversity of the samples was processed with Vegan (version 2.4-6) in R package. The top 100 most abundance clades in each sample were selected to calculate the “Bray-Curtis” distance and the similarity between samples (**Figure S3**). The linear discriminant effect size (LEfSe) analysis was performed to find features (taxa) differentially represented between groups (48).

The Short Oligonucleotide Analysis Package(SOAP2, version 2.21) (49) was used to do the alignment and retain the unique mapped reads to do the downstream analysis. The relative abundance of these (super)contigs or genomes was calculated based on the number of aligned reads normalized by the (super)contig’s or genome’s size. The integrated non-redundant gene catalog database about the human gut microbiome was used to do the function analysis (50) with the Kyoto Encyclopedia of Genes and Genomes (KEGG) database, the Carbohydrate-Active Enzymes database (CAZy), the Pathogen–Host Interactions database (PHI-base) and the Gut-Brain Modules (GBMs) as described in previously published articles (51–53).

Other statistical analyses were performed using Statistical Package for the Social Science version 19.0 (SPSS Inc., Chicago, Illinois) and GraphPad Prism version 5.0 (GraphPad Software Inc., San Diego, CA). Continuous data were checked for normal distribution using the Shapiro-Wilk test first. Unpaired t test or non-parametric test (for those data that were not normally distributed) was used for comparison between groups. The Spearman or Pearson correlation test was applied to explore the correlation among autistic symptoms, gut microbiota, and functional categorization. The principal component analysis (PCA), orthogonal partial least-squares discriminate analysis (OPLS-DA) and the multivariate receiver operator curve (ROC) analysis were carried out using the methods as described in the protocol (54). For all tests, a value of *p*<0.05 (two-tailed) was considered statistically significant. False discovery rates (FDR) were controlled at 0.05 for multiple testing using the Benjamini and Hochberg method.

## Results

### Characteristics of the enrolled participants

A total of 105 individuals met the inclusion criteria were recruited, and the top and bottom quarter of the participants were selected for questionnaires and fecal microbiota analyses based on their IFN-γ level derived from γδT cells. Since some of the participants were outpatients who can only spare a little time with our team, and some of the children may not defecate within these few days, not all of them have time to complete the behavioral symptoms assessment or have their fecal sample successfully collected. Those who completed all the questionnaires and fecal sample collection for metagenomic array constitute the final study samples in the present study.

Demographics of the participants were summarized in **Table 1**. The two groups were well matched for chronological age and sex composition. The incidences of gastrointestinal symptoms such as diarrhea and constipation showed no statistical difference between groups. As expected, levels of IFN-γ derived from γδT cell were much higher in autistic children in the IFN-γ-High group as compared to the IFN-γ-Low group.

**Table 1:** Characteristics of the enrolled participants.

	IFN-γ-Low	IFN-γ-High	*p*
**n**	17	21	
**Age(y) [mean ± SEM(range)]**	4.78 ± 0.36 (3.15-8.47)	4.74 ± 0.36 (3.02-8.54)	0.949
Gender			
Male (%)	14 (82.35%)	19 (90.48%)	0.640
Female (%)	3 (17.65%)	2 (9.52%)	
GI symptoms			
Diarrhea (%)	1 (5.88%)	3 (14.29%)	0.613
Constipation (%)	8 (47.06%)	6 (28.57%)	0.318
Diarrhea & Constipation^#^ (%)	3 (17.65%)	5 (23.81%)	0.697
**IFN-γ [mean ± SEM(range)]**	0.80 ± 0.10 (0.09-1.32)	5.73 ± 0.48 (3.83-11.88)	<0.001

^#^ The term “Diarrhea & Constipation” indicates children with alternative symptoms of diarrhea and constipation.

Data are presented as mean ± standard error of mean (SEM) for Age and IFN-γ.

### Differences in the severity of autistic behavioral symptoms between groups

Preliminary analysis of IFN-γ levels vs ASD severity indicated in recent clinical records (graded as mild, moderate or severe) suggests a rather skewed distribution (**Figure S1**).

**Figure 1:**
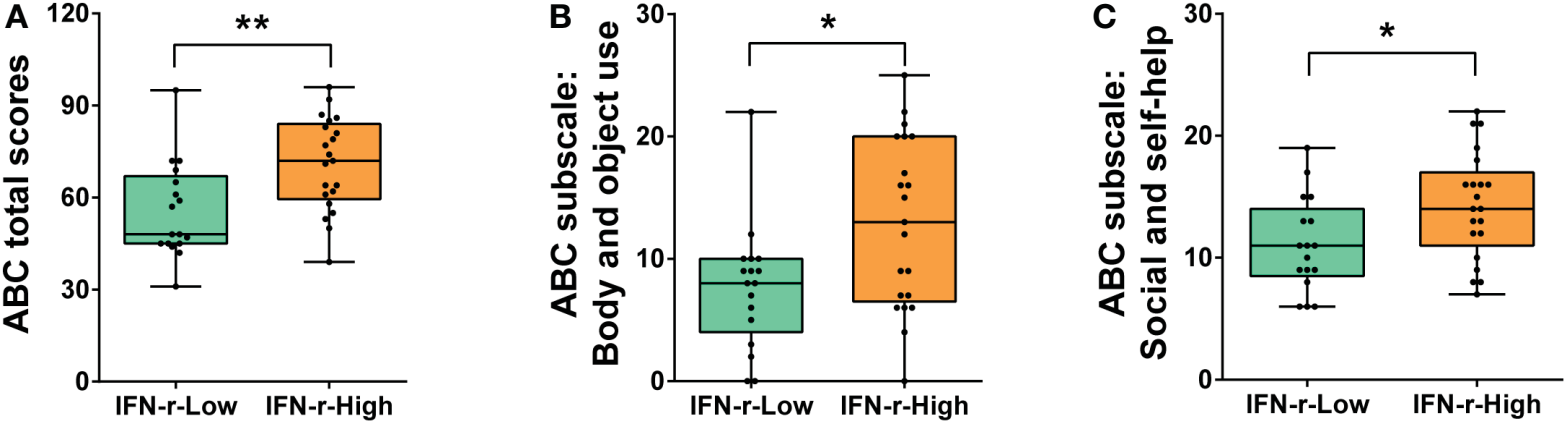
Differences of ABC total and subscales scores between the IFN-γ-Low and IFN-γ-High groups. **(A)** ABC total scores; **(B)** Scores of ABC subscale III: Body and object use; **(C)** Scores of ABC subscale V: Social and self-help. The horizontal line and the box indicate the median and the interquartile range (IQR), and the whisker spans the minimum to maximum. ^*^ *p*<0.05, ^**^ *p*<0.01.

Significant differences in ABC metrics for severity of autistic behavioral symptoms were found between the IFN-γ-High and IFN-γ-Low groups. Children in the IFN-γ-High group had significantly higher ABC total scores (Median: 72.0, interquartile range (IQR): 59.5~84.0) than those in the IFN-γ-Low group (48.0, IQR 45.0~67.0, *p*<0.01) (**Figure 1A**).

The *post hoc* analyses were conducted on the subscales scores (**Table 2**), and scores of two subscales in ABC (Body and object use, Social and self-help) demonstrated statistical differences between the two groups (**Figures 1B, C**), indicating that the related symptoms of those children in the IFN-γ-High group were much severe than those in the IFN-γ-Low groups.

**Table 2:** Comparison of ABC and ATEC subscales scores between groups.

	Group		*Z*	*p*
	IFN-r-Low	IFN-r- High		
ABC subscales				
I. Sensory	12.0(7.0,18.0)	16.0(10.0,18.0)	1.263	0.207
II. Relating	11.0(8.0,16.5)	16.0(11.0,19.0)	1.503	0.133
III. Body and object use	8.0(4.0,10.0)	13.0(6.5,20.0)	2.061	0.039^*^
IV. Language	12.0(9.0,15.0)	14.0(10.0,19.0)	0.943	0.346
V. Social and self-help	11.0(8.5,14.0)	14.0(11.0,17.0)	2.179	0.029^*^
ATEC subscales				
I. Speech/Language/Communication	12.0(6.0,15.5)	20.5(12.0,23.5)	2.261	0.024^*^
II. Sociability	22.0(18.5,30.0)	23.0(18.0,28.5)	0.28	0.78
III. Sensory/Cognitive Awareness	19.0(13.5,25.0)	20.0(17.0,26.0)	0.883	0.377
IV. Health/Physical/Behavior	28.0(19.5,30.5)	23.0(20.0,36.5)	0.353	0.724

ABC, Autism Behavior Checklist; ATEC, Autism Treatment Evaluation Checklist.

Data are presented as median (P25, P75). ^*^p<0.05.

**Figure 2:**
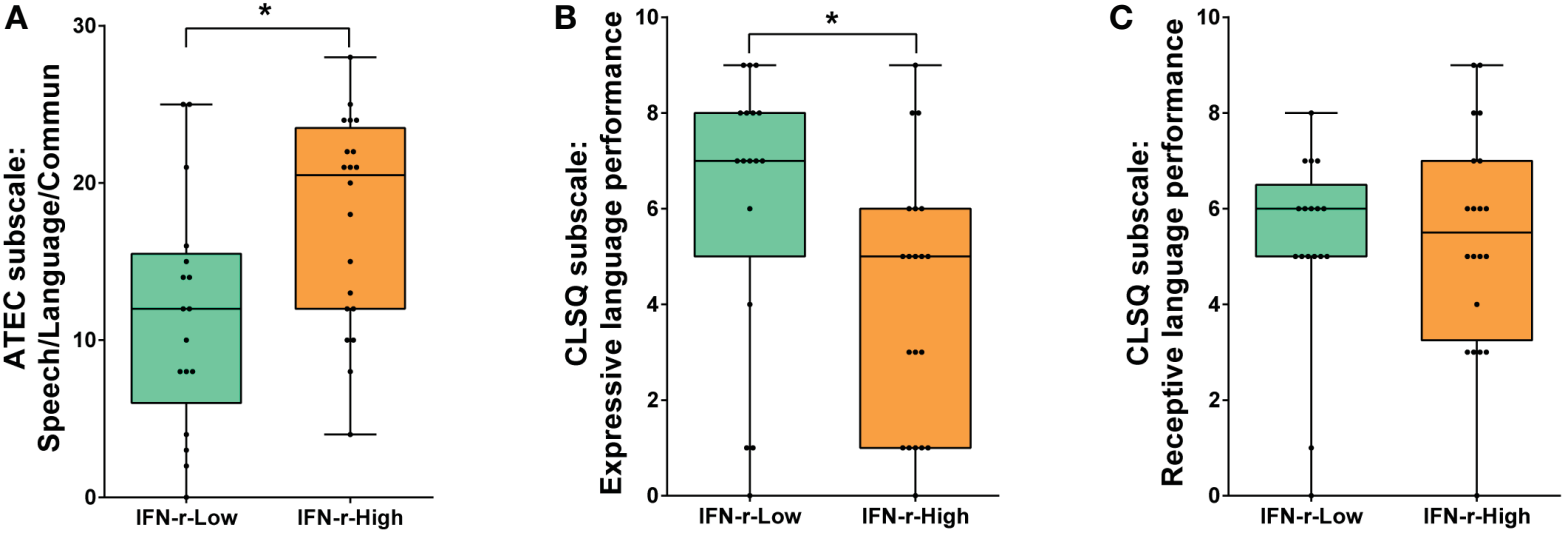
Comparison of language function scores between the IFN-γ-Low and IFN-γ-High groups. **(A)** Scores of ATEC subscale I: Speech/Language/Communication; **(B)** Scores of CLSQ expressive language performance; **(C)** Scores of CLSQ receptive language performance. The horizontal line and the box indicate the median and the interquartile range (IQR), and the whisker spans the minimum to maximum. ^*^ *p*<0.05.

There was no statistical difference in ATEC total scores between groups (88.5, IQR 70.5~104.8 in IFN-γ-High group vs. 82.0, IQR 61.5~93.5 in IFN-γ-Low group, *p*>0.05). However, the *post hoc* analyses of the subscales scores (**Table 2**) showed that scores of the Speech/Language/Communication subscale in ATEC demonstrated statistical difference (**Figure 2A**), indicating the language function was much more impaired in the IFN-γ-High group.

In order to further evaluate the children’s expressive and receptive language performance respectively, the Clinical Language Status Questionnaire (CLSQ) was applied. As is shown in **Figure 2B**, children in the IFN-γ-High group got lower expressive language performance scores (5, IQR1~6) than those in the IFN-γ-Low group (7, IQR 5~8, *p*<0.05). However, scores of receptive language performance showed no difference between the two groups **(** **Figure 2C**).

### Differences in the fecal microbiota composition between groups

**Figure 3:**
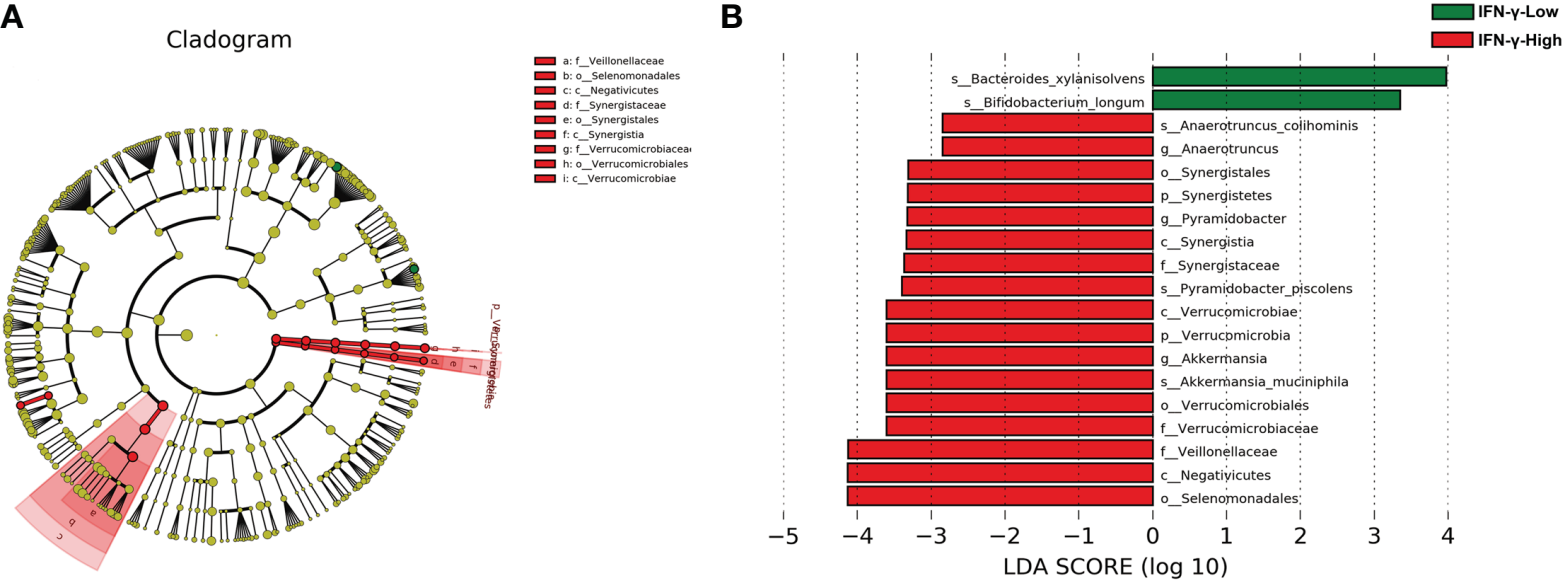
Cladograms generated by LEfSe and LDA scores for bacterial taxa differentially abundant between groups. **(A)** Cladograms indicating differences in the bacterial taxa between the IFN-γ-Low and IFN-γ-High group. Green and red nodes indicate taxa that were enriched in the IFN-γ-Low group and the IFN-γ-High group, respectively. **(B)** LDA scores for differentially abundant bacterial taxa. Only taxa having a *p*<0.01 and LDA>2 are shown. Positive LDA scores indicate the taxa enriched in the IFN-γ-Low (IFN-γ-L) group (green), while negative LDA scores indicate the taxa enriched in the IFN-γ-High (IFN-γ-H) group (red), respectively.

There was no significant difference in the alpha-diversity of the fecal microbiota between the two groups (**Figure S2**). The Bray–Curtis dissimilarity revealed no significant difference between the two groups (**Figure S3**, PERMANOVA, *r* ^2 =^ 0.0276, *p*= 0.398). The LEfSe method was used to determine the taxa at different taxonomic levels which were enriched in the IFN-γ-High and IFN-γ-Low groups (**Figure 3**). Results of the LEfSe analysis revealed underrepresentation of *Bacteroides xylanisolvens* and *Bifidobacterium longum* in the IFN-γ-High group (*p*<0.01, Wilcoxon rank-sum test; LDA>3.0). Overrepresentation of *several phylotypes were also found in the* IFN-γ-High group, and *Selenomonadales*, *Negatiyicutes* and *Veillonellaceae* were the top 3 enriched phylotypes with LDA<-4.0.

### Differences in functional profiles from the metagenomic data between groups

**Figure 4:**
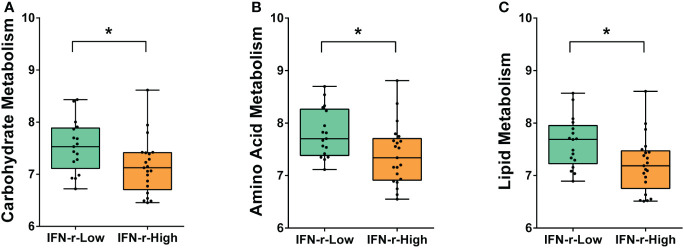
Differences of enriched KEGG orthologues markers between the IFN-γ-Low and IFN-γ-High groups. The values of the points represent the relative abundances of the KEGG orthologues markers related to **(A)** carbohydrate metabolism, **(B)** amino acid metabolism and **(C)** lipid metabolism. The horizontal line and the box indicate the median and the interquartile range (IQR), and the whisker spans the minimum to maximum. ^*^ *p*<0.05.

From the metagenomic data, the KEGG orthologues markers that were different between the IFN-γ-Low and IFN-γ-High groups were analyzed. The relative abundance of the KEGG orthologues markers related to amino acid metabolism, carbohydrate metabolism and lipid metabolism were found to be decreased in the IFN-γ-High group as compared to the IFN-γ-Low group with values of *p*<0.05 (**Figure 4**). And the reported differences remained significant after applying the FDR.

**Figure 5:**
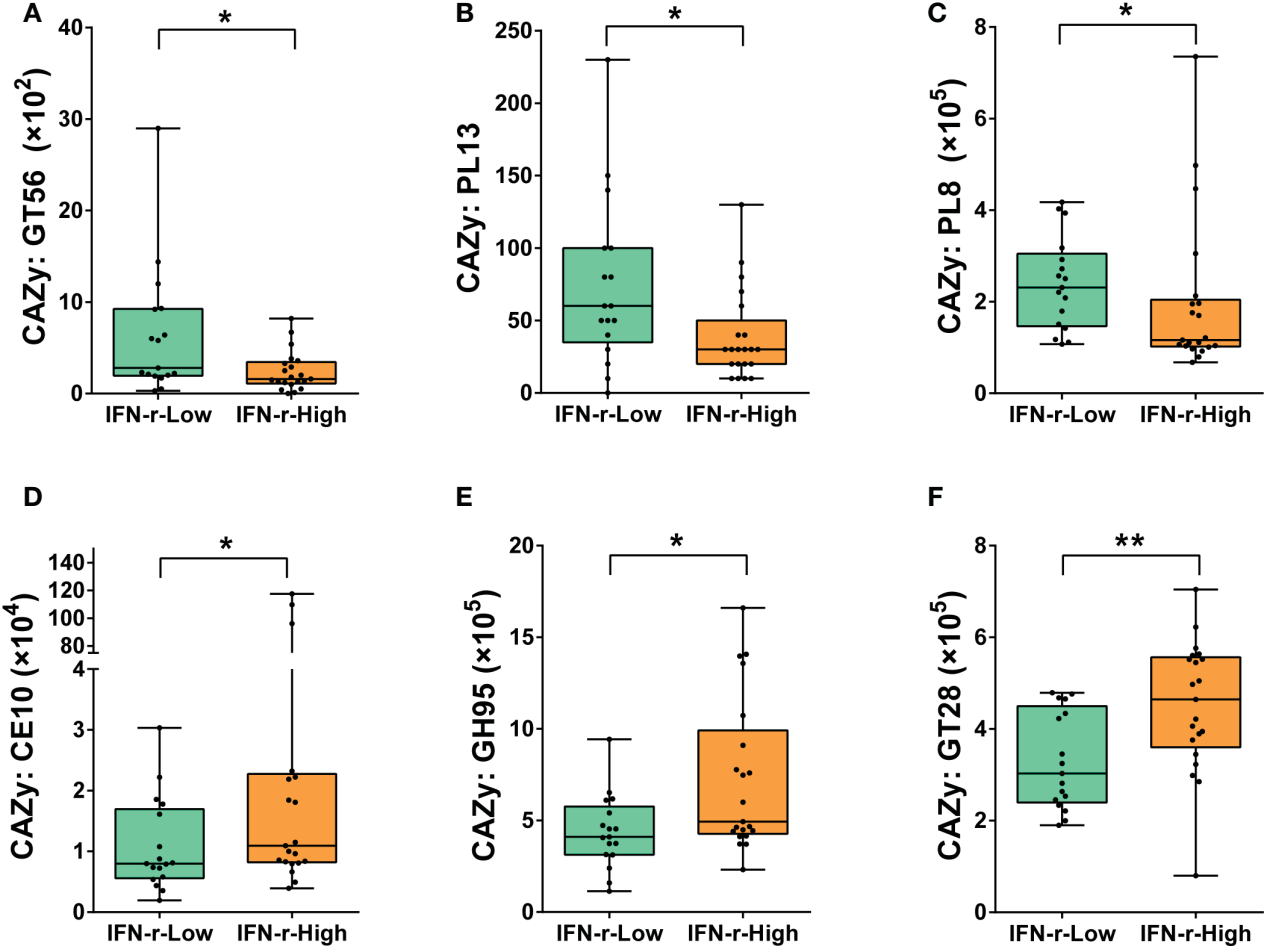
Differences of the abundances of genes encoding carbohydrate-active enzymes (CAZymes) in the fecal microbiome between the IFN-γ-Low and IFN-γ-High groups. The values of the points represent the relative abundances of genes encoding **(A)** GlycosylTransferase Family 56 (GT56), **(B)** Polysaccharide Lyase Family 13 (PL13) and **(C)** Polysaccharide Lyase Family 8 (PL8), **(D)** Carbohydrate Esterase Family 10 (CE10), **(E)** Glycoside Hydrolase Family 95 (GH95) and **(F)** GlycosylTransferase Family 28 (GT28). The horizontal line and the box indicate the median and the interquartile range (IQR), and the whisker spans the minimum to maximum. ^*^ *p*<0.05, ^**^ *p*<0.01.

In order to further explore the possible mechanisms underlying the differences relating carbohydrate metabolism between groups, the abundances of genes encoding carbohydrate-active enzymes (CAZymes) in the fecal microbiome were quantified. CAZymes were annotated by their family as defined in the CAZy database. Significant differences after applying the FDR in the abundances of genes encoding six CAZymes families were found between the two groups. For children in the IFN-γ-High group, the relative abundances of genes encoding GlycosylTransferase Family 56 (GT56), Polysaccharide Lyase Family 13 (PL13) and Polysaccharide Lyase Family 8 (PL8) was lower, while the relative abundances of genes encoding Carbohydrate Esterase Family 10 (CE10), Glycoside Hydrolase Family 95 (GH95) and GlycosylTransferase Family 28 (GT28) was higher as compared to those in the IFN-γ-Low group (**Figure 5**).

**Figure 6:**
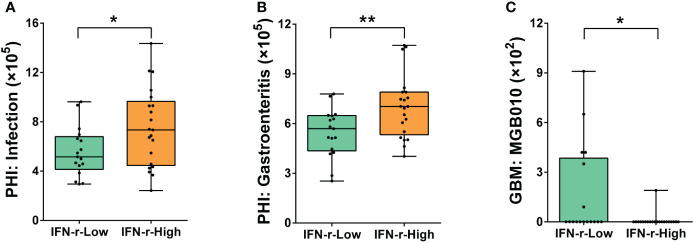
Differences of the abundances of genes related to pathogen-host interactions and gut–brain modules in the fecal microbiome between the IFN-γ-Low and IFN-γ-High groups. The values of the points represent the relative abundances of genes related to **(A)** infection (Pathogen gene: *purT*, Host species: *Homo sapiens*), **(B)** gastroenteritis (Pathogen gene: *flhF*, Host species: *Homo sapiens*) and **(C)** gut–brain module MGB010 associated with histamine degradation. The horizontal line and the box indicate the median and the interquartile range (IQR), and the whisker spans the minimum to maximum. ^*^ *p*<0.05, ^**^ *p*<0.01.

The PHI-base phenotypes related to infection (Pathogen gene: *purT*, Host species: *Homo sapiens*) and gastroenteritis (Pathogen gene: *flhF*, Host species: *Homo sapiens*) were found to be significantly enriched in the IFN-γ-High group (**Figures 6A, B**). Additionally, underrepresentation of one gut–brain module (MGB010) associated with histamine degradation was also found in the IFN-γ-High group (**Figure 6C**).

### The correlation analysis

**Figure 7:**
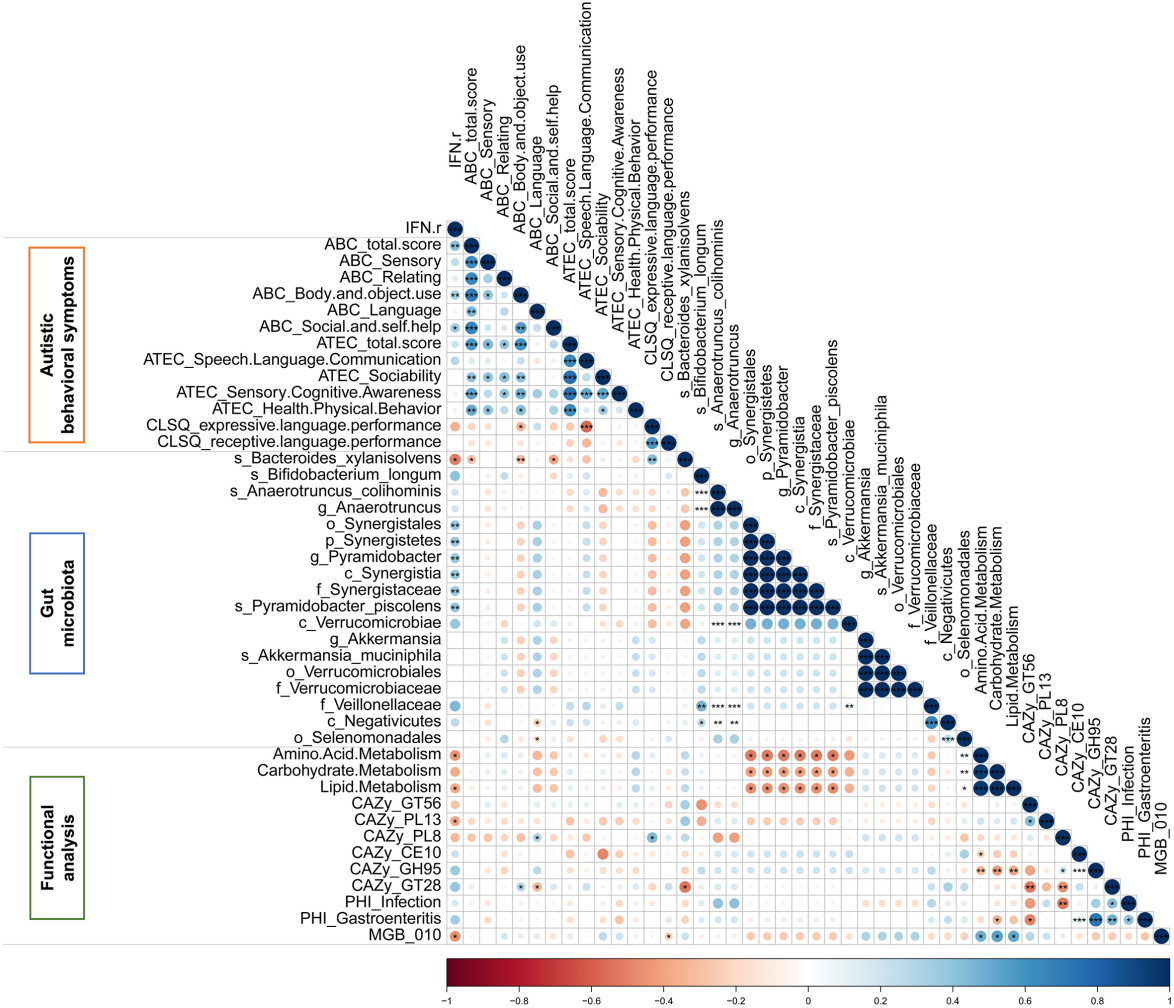
The Spearman correlation matrix among IFN-γ, autistic behavioral symptoms, gut microbiota and functional modules which were significant in autistic children. Color intensity reflects Spearman correlation coefficient. ^*^ *p*<0.05, ^**^ *p*<0.01, ^***^ *p*<0.001.

The Spearman correlation analysis was applied to explore the relationship among IFN-γ, autistic behavioral symptoms, gut microbiota and functional modules which were significant in univariate analysis. As is shown in the matrix in **Figure 7**, the relative abundance of *Bacteroides xylanisolvens*, which was enriched in the IFN-γ-Low group, was negatively correlated with IFN-γ level (*rho*=-0.434, *p*<0.05), ABC total and subscales (Body and object use, Social and self-help) scores (all *p*<0.05), and the relative abundance of GlycosylTransferase Family 28 (GT28) (*rho*=-0.535, *p*<0.05), and positively correlated with CLSQ expressive language performance scores (*rho*=0.353, *p*<0.01). Additionally, the relative abundance of GlycosylTransferase Family 28 (GT28) was negatively correlated with ABC language scores (*rho*=-0.326, *p*<0.05), while the relative abundance of Polysaccharide Lyase Family 8 (PL8) was positively correlated with ABC language score (*rho*=0.303, *p*<0.05). Among these above correlations, only the correlation between the relative abundance of *Bacteroides xylanisolvens* and CLSQ expressive language performance scores remained significant after applying the FDR.

### Multivariate analysis and potential discriminating features analysis

**Figure 8:**
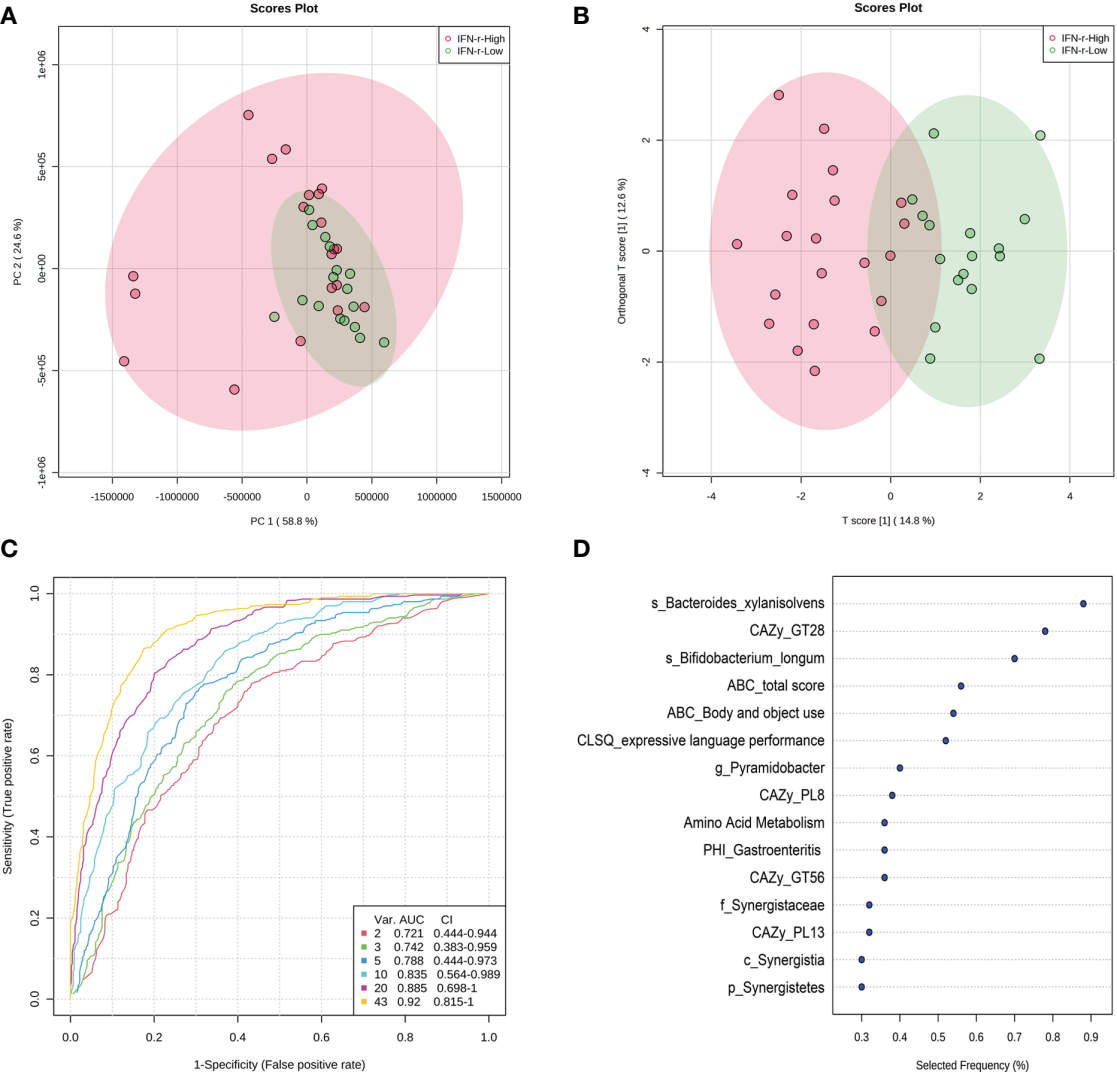
Scores plot of multivariate analysis and potential discriminating features analysis using random forests algorithm. **(A)** Principal component analysis (PCA) scores plot and **(B)** Orthogonal partial least-squares discriminate analysis (OPLS-DA) scores plot of ASD children in the IFN-γ-Low (green) and IFN-γ-High (red) groups. Each point represents the score of a single individual. The shaded areas indicate the 95% confidence ellipse regions for each group. **(C)** ROC curves from different multivariate models using different number of features. **(D)** The top 10 significant discriminating features ranked based on their frequencies of being selected during cross validation.

Since univariate approaches ignore the correlations among variables as demonstrated in **Figure 7**, multivariate analyses were applied because these methods simultaneously take all variables into consideration. The principal component analysis (PCA) scores plot revealed that samples in the IFN-γ-Low group were more concentrated as compared to the scattered pattern of the IFN-γ-High group (**Figure 8A**). And as is shown in **Figure 8B**, the scores plot constructed using orthogonal partial least-squares discriminate analysis (OPLS-DA) revealed relatively good separation between the IFN-γ-Low and IFN-γ-High groups (Q2 = 0.469, *p*<0.05; R2Y=0.726, *p*<0.05).

The algorithm of the random forests was used to perform potential discriminating features analysis. The Monte-Carlo cross validation (MCCV) was applied to identify models with good performance. In each MCCV, two thirds (2/3) of the samples are used to evaluate the feature importance. The top 2, 3, 5, 10… important features are then used to build classification models which is validated on the 1/3 the samples that were left out. The procedure were repeated multiple times to calculate the performance and confidence interval (CI) of each model. Based on the cross validation, the multivariate models using 10 variables achieved an AUC of 0.835 (**Figure 8C**). The top 10 significant discriminating features ranked based on their frequencies of being selected during cross validation are listed in **Figure 8D**.

## Discussion

In the present study, a cohort of 105 ASD children were recruited and ranked based on their IFN-γ levels derived from γδT cells. The top 25% and bottom 25% of the participants were selected which constituted the final two groups, respectively. Our results demonstrated that autistic behavioral symptoms of children in the IFN-γ-high group were more severe, especially in the body and object use, social and self-help, and expressive language performance domains. The LEfSe analysis of gut microbiota revealed some bacterial taxa differentially abundant between groups. Decreased metabolism function of carbohydrate, amino acid and lipid in gut microbiota were found in the IFN-γ-high group. Additional functional profiles analyses also revealed significant differences in the abundances of genes encoding carbohydrate-active enzymes between groups. And enriched phenotypes related to infection and gastroenteritis and underrepresentation of one gut–brain module associated with histamine degradation were also found in the IFN-γ-High group. Results of multivariate analyses revealed relatively good separation between the two groups and suggest that IFN-γ could serve as a potential candidate biomarker to subtype ASD individuals into more homogeneous subtypes.

Currently, the diagnosis of ASD is still made mainly based on behavioral symptoms (1). And the concept of spectrum suggests that individuals with ASD may present with diverse sets of symptoms that vary widely from one individual to another (1, 55). The symptom diversity may be caused by many different factors, and this heterogeneity brings about great difficulty for researchers to elucidate the anticipated etiology or risk factors for ASD, because it would not be expected that a same etiological factor would explain two vastly different phenotypes (56, 57). It has now been well recognized that researchers should subtype these individuals within the spectrum to reduce the diversity and use a more homogeneous subtype to study the biological mechanism and explore effective treatment strategies (58). Previous subtyping strategies are mostly defined by some particular symptom characteristics, such as social behavior or language ability (6, 7, 9, 10). Another feasible approach is using biomedical features to stratify samples to reduce heterogeneity and produce subgroups which are more likely to share a more similar phenotype and etiology (11, 12, 59). Compared to the behavioral symptom characteristics, using biomedical features as subtyping indicators has some advantages, because they are more objective and easily to measure, more directly to indicate the possible mechanisms underlying the associated heterogeneity, and could also provide useful information to further explore the potential targets to facilitate the development of individualized biomedical therapy strategies for certain ASD subtypes.

Immunological involvement in the pathophysiology of certain subtypes of ASD has long been hypothesized and accumulated results from both clinical and animal research have identified the associations between immunologic function abnormalities and ASD (12, 21–24, 60–62). Moreover, clinical trials using immune-modulating or anti-inflammatory drugs in individuals with ASD also yield promising results, and the treatment responses were especially better for those with immunological or gastrointestinal disturbances (12, 63–66). Results of these previous studies suggest that biological characteristics relating to immune function may serve as potential biomarkers to reduce the heterogeneity in ASD and to improve the prediction of response to certain biomedical treatments (12).

In the present study, we choose IFN-γ derived from γδT cells as subtyping biomarkers, because γδT cell intrinsically combines innate immunity and adaptive immunity and plays important roles in inflammatory and autoimmune diseases, which were found to be more prevalent in ASD individuals (21–24, 67). And results of previous studies also indicated IFN-γ might play a role in the progression and exacerbation of autistic symptoms (28–31). Changes of INF-γ levels have been found in blood samples and brain tissues of ASD subjects, and animal studies also confirmed upregulation of INF-γ in animals with autistic-like behaviors (31, 68, 69). It has been demonstrated that plasma levels of INF-γ correlated positively with plasma nitric oxide measures in ASD group and the higher NO production in ASD children may be secondary to IFN-γ mediated up-regulation of the inducible nitric oxide synthase (iNOS) (70). INF-γ may interact with gut microbiota and PBMCs taken from ASD subjects produced elevated levels of IFN-γ against common dietary proteins (71). High levels of INF-γ were also associated with a reduction in glucocorticoid receptor (72), which might result in excessive circulation of glucocorticoid, and the excessive glucocorticoid are well-known as neurotoxins (73). Although the direct solid evidence is still lacking, these findings support the hypothesis that INF-γ may play a role in the pathologic mechanism of ASD.

However, results of the INF-γ levels in ASD from the previous studies were not always consistent. Both higher and lower levels of INF-γ have been found in blood samples and PBMCs of ASD [see the summarized results in the excellent systematic reviews (74, 75)]. In our clinical practice, we also find that a great heterogenicity exists in the INF-γ levels in ASD. Levels of INF-γ are very high in a portion of ASD children, and their behavioral symptoms seems to be different from other ASD children. So, we hypothesized that within the heterogeneous broad spectrum of ASD, those ASD children with high INF-γ levels may represent a subgroup whose autistic symptoms and gut microbiota composition may be different from others. And the results of the present study turned out to support our initial hypothesis.

When comparing the autistic behavioral symptoms between the two subgroups selected based on levels of IFN-γ derived from γδT cells, children with higher levels of IFN-γ got significantly higher scores in ABC, especially for the body and object use subscale and the social and self-help subscale. Additionally, children in this group also got higher scores in the speech/language/communication subscale in ATEC. Since there were some discrepancies as assessed by ABC and ATEC questionnaires in the language domain, and the expressive and receptive language abilities were evaluated with different weights but calculated as a whole in these two questionnaires (44, 45), the CLSQ was used to further assess children’s expressive and receptive language performance respectively (46). The results revealed that only the expressive language performance was significantly impaired in the IFN-γ-High group. All these results suggest that autistic behavioral symptoms were different between the IFN-γ-High and IFN-γ-Low groups, and children with higher levels of IFN-γ may suffer from more severe symptoms of ASD.

Since there exist intense interactions between gut microbiota and immune function, and alterations of gut-immune-brain axis has been suggested to act critical roles in the pathogenesis of ASD (32–35, 41, 42), differences in gut microbiota composition between the two groups were also analyzed. The most significant characteristic difference between the two subgroups is that *Negativicutes*, *Selenomonadales* and *Veillonellaceae* were more enriched in the IFN-γ-High group, with the LDA score less than -4. Different abundances of these three bacterial taxa were also found between autistic and neurotypical subjects in several other independent studies (23, 76, 77). Indeed, the family *Veillonellaceae* belongs to the order *Selenomonadales* within the class *Negativicutes*, and they are all members of the phylum *Firmicutes* (78). Species of *Firmicutes* could upregulate IFN-γ production and significant increased ratio of *Firmicutes*/*Bacteroidetes* has been reported associated with not only with ASD (77, 79), but also with other conditions that were found to be more prevalent within ASD subjects, such as obesity and diabetes (24, 80, 81).

The relative abundances of the species of *Akkermansia muciniphila*, *Pyramidobacter piscolens*, and *Anaerotruncus colihominis* were also found to be more enriched in the faces of ASD children in the IFN-γ-High group. *Akkermansia muciniphila* is a mucin-degrading bacterium, which has been suggested to play a role in inflammation and gut permeability (82, 83). Lower relative abundances of *Akkermansia muciniphila* has been found in feces of autistic children, which might reflect an indirect evidence of a thinner gastrointestinal mucus barrier in ASD children (83). Interestingly, there are also studies that found *Akkermansia* was present at higher relative abundances in feces of ASD individuals (76, 84), or even at very high levels (up to 59%) in several autistic individuals (23). Results from these previous studies suggest that great diversity in the abundances of *Akkermansia muciniphila* may exist among different ASD individuals. In the present study, we found that within the spectrum of autism, there do exist significant differences in the relative abundances of *Akkermansia muciniphila* between ASD children in the IFN-γ-High and the IFN-γ-Low groups. *Pyramidobacter piscolens* is one of the members of the phylum *Synergistetes*. It was first isolated from human oral cavity (85) and is related to oral dysbiosis, which may result in periodontal diseases and abscess (86). Oral dysbiosis and these oral health conditions are also found to be more common in ASD children (87). Further studies revealed that *Pyramidobacter piscolens* could also be cultured from small intestine abscess, and it is now considered that *Pyramidobacter piscolens* is part of the commensal human microflora which plays a functional role but may also act as opportunistic pathogens (88). Additionally, *Pyramidobacter piscolens* is one of the core species which can regulate lipid deposition (89) and may influence blood glucose metabolism (90). *Anaerotruncus colihominis* belongs to phylum *Firmicutes*. It is a short-chain fatty acids (SCFA) producing species which is presumed to be anti-inflammatory and is related to autoimmunity (91–94). The abundance of *Anaerotruncus colihominis* was found to be negatively associated with cognitive function scores in patients with Alzheimer’s disease (95). Significant lower abundances of *Anaerotruncus colihominis* has also been found in patients with rheumatoid arthritis (RA) (94), and a number of clinical and basic studies have demonstrated roles of IFN-γ in the pathogenesis of RA (96). It has also been reported that *Anaerotruncus colihominis* possesses the ability to produce acetic and butyric acids (91), which could have a role in regulating gut epithelial barrier function and play possible roles in ASD (97, 98). Although both of the *Pyramidobacter piscolens* and *Anaerotruncus colihominis* play functional roles in the metabolism of bioactive compounds which is perturbed in ASD, studies of the direct roles of the two species in the pathophysiology of ASD is rare. Our results demonstrated that there were significant differences in the relative abundances of *Pyramidobacter piscolens* and *Anaerotruncus colihominis* between ASD children in the IFN-γ-High and the IFN-γ-Low groups, and the biological significance of these findings warrants further research.

The results of the LEfSe analysis also revealed underrepresentation of *Bacteroides xylanisolvens* and *Bifidobacterium longum* in the IFN-γ-High group. These two bacterial species are both non-pathogenic and process many probiotic qualities (99–101). *Bacteroides xylanisolvens* belongs to the second most abundant genus *Bacteroides* in the human intestine and they can break down many sugars including dietary fiber and xylan (102, 103). It has been demonstrated that some strains of *Bacteroides* could modulate the function of innate immune system (104) and have the potential to relieve some behavioral and physiological abnormalities associated with ASD (105, 106). *Bifidobacterium longum* is considered to be one of the earliest colonizers of the gastrointestinal tract in infants (107). The domination of *Bifidobacterium* in infant’s gastrointestinal tract could hinder pathogenic organisms’ colonization through antimicrobial activity and competitive exclusion manners (108). *Bifidobacterium longum* could also serve as a scavenger because it metabolizes a large variety of substrates including bile salts, human milk oligosaccharide and some other complex oligosaccharides (107, 109, 110). The efficacy of *Bifidobacterium longum* in regulating immune (including its ability to suppress the expression of IFN-γ *in vivo*) and central nervous system functions and alleviating psychiatric disorder-related behaviors including ASD and obsessive-compulsive disorder has also been demonstrated (100, 101, 111). It is worth mentioning that *Bacteroides* and *Bifidobacterium* species were also found to be depleted in ASD children in other independent cohort studies (76, 83, 112, 113). Associations between gut microbiota and ASD certainly warrant further studies to elucidate a causation role in the pathogenesis of ASD. However, the consistency of these results across different ethnic groups using different sequencing and assay methods, together with their efficacy in alleviating autistic symptoms, strongly suggest that the loss of representation of these bacterial taxa is very robust and may be tightly associated with the pathophysiology of ASD.

For the predicted KEGG pathway analysis results, we found that the IFN-γ-High group was less enriched in pathways related to amino acid metabolism, carbohydrate metabolism and lipid metabolism. As key partners involved in the maintenance of human physiology and health, gut microbes influence greatly on host metabolism and help balance important vital functions such as food digestion and nutrient bioavailability for the host (114). The relatively depleted pathway orthologues markers related to metabolism of amino acid, lipid and carbohydrate in the IFN-γ-High group suggested that children in this subgroup may have higher risks of suffering from more sever metabolic dysfunction. Indeed, a great quantity of work has shown that children with ASD have perturbed metabolism as compared to neurotypical children (112, 115–123). For the amino acid metabolism, altered amino acid profile has been found in blood plasma (116, 117), urine (118, 119) and fecal (112) samples collected from ASD individuals. And it was postulated that gut microbial metabolism of phenylalanine and tyrosine may be involved in the pathogenesis of autism (120). Impaired carbohydrate digestion (121) and lipid metabolism (122) were also found in ASD individuals. The abundance of affected bacterial phylotypes in the intestines or duodenum of ASD individuals was found to be associated with expression levels of disaccharidases and transporters, which is important for carbohydrate digestion and transport (121, 123). Since *Bacteroides* spp. and *Bifidobacterium* spp. are specialized as primary and secondary degraders in the metabolism of complex carbohydrates (124), the depleted species of *Bacteroides* and *Bifidobacterium* in the IFN-γ-High group may impact the carbohydrate metabolism capability. Additionally, as is demonstrated in this study, the abundances of genes encoding six families of carbohydrate-active enzymes in the fecal microbiome were significantly different between the IFN-γ-High and IFN-γ-Low groups, this may partly explain the possible mechanisms underlying the differences relating carbohydrate metabolism between the two groups. Furthermore, some bacterial species possess the ability to ferment dietary carbohydrates into the production of short chain fatty acids (SCFAs) (125). SCFAs can readily cross the gut–blood and blood–brain barriers and induce widespread effects on gut and brain *via* impact on epithelial barrier integrity, neurotransmitter synthesis and immune modulation (126–128). Since some of the metabolites such as Omega-6 (n-6) and Omega-3 (n-3) polyunsaturated fatty acids (PUFA) are essential nutrients for brain development and function, these metabolic alterations may be associated with the severity of autistic symptom (122, 129, 130). All these results further support the notion that ASD is a pervasive developmental disorder with multisystem dysfunction and metabolic disturbance.

Functional profiles analyses from the metagenomic data in our study also revealed that the abundances of genes related to infection (Pathogen gene: *purT*, Host species: *Homo sapiens*) and gastroenteritis (Pathogen gene: *flhF*, Host species: *Homo sapiens*) were significantly enriched in the IFN-γ-High group. Gastrointestinal disorders are one of the most common medical conditions that are comorbid with ASD, and these comorbidities can cause greater severity in autistic symptoms (131). The results from our study further suggest that children in the IFN-γ-High group may suffer from higher incidence or severity of infection and gastroenteritis, but these results need to be further validated with medical examination. Another significant difference is the underrepresentation of the gut–brain module (MGB010) associated with histamine degradation in the IFN-γ-High group. Altered expression of histamine signaling genes has been found in ASD populations (132), and antagonism of histamine receptors could reduce autistic behavioral symptoms in ASD individuals and several relevant animal models (132–135). Moreover, histamine receptor antagonists can suppress IFN-γ production (136), while IFN-γ can also modulate histamine-induced IL-6 and IL-8 production (137). Our data suggests the histamine degradation capability in fecal microbiota were much more impaired in children with higher levels of IFN-γ, and the decreased capability of histamine degradation may partly affect the autistic behavioral symptoms in ASD children.

For the correlation analysis, the relative abundance of *Bacteroides xylanisolvens* showed most significant relationships with not only several autistic behavioral characteristics, but also with one of the carbohydrate-active enzymes families (GT28). Additionally, *Bacteroides xylanisolvens* was the top discriminating features in the multivariate models using the random forests algorithm, suggesting its importance in the separation of the two groups. Our results of the multivariate analysis indicate that although both of the two groups are within the spectrum, they can be separated using the IFN-γ as indicator to obtain subtypes with more similar features. Based on the cross validation, the ROC curves built using 10 variables achieved an AUC of 0.835. In this study, the ROC curves were generated by MCCV using balanced sub-sampling. In each MCCV, two thirds (2/3) of the samples were used to evaluate the feature importance. The top 2, 3, 5, 10… important features were then used to build classification models which were validated on the 1/3 samples that were left out (54). Since more variables consistently leads to better prediction, and due to the relatively small sample size in this study, there exists a risk of overfitting. Therefore, it is important to evaluate the models with a large number of samples to estimate their generalizability with high confidence.

Since INF-γ level varies widely within the heterogeneous broad spectrum of ASD, and as is shown in our study, the behavioral symptoms, gut microbiota composition and some metabolic features of ASD children in the INF-γ-High group were different from those in the INF-γ-Low group, utilization of this information to segregate ASD children into different subgroups will greatly facilitate the pathophysiology study of a more homogeneous clusters of ASD in the future. Additionally, although there still lack of solid evidence, several anti-inflammatory compounds (such as Palmitoylethanolamide, celecoxib, flavonoid luteolin) have been studied to investigate their effect as an adjunctive therapy in improving behavioral symptoms in autistic individuals (64, 138, 139). Since INF-γ is an important pro-inflammatory cytokine involved in ASD but varies widely within the heterogeneous spectrum, we believe that using these anti-inflammatory drugs in ASD subgroup with high INF-γ levels will yield more promising and consistent results.

As a preliminary study, there are several limitations ought to be mentioned. Firstly, only children with ASD were enrolled in this study, lacking typically developing children as controls, and the comparison of these obtained results with a control group can be informative. Secondly, the sample size in this study is relatively small, which may decrease the statistical power, and there exists a risk of overfitting for the MCCV model. Results of this study need to be validated in an independent larger cohort. Thirdly, additional risk factors (such as having a close relative with ASD, very low birth weight, and complications at delivery) were not collected from the participants in this study. The results should be interpreted with caution due to the observational nature of the present study. Also, consistent with the sex ratio of ASD, participants were mostly males, which limited the analyses of sex differences. Finally, only IFN-γ was measured in this study without testing other cytokines such as interleukin and TNF, which limits the ability to explore the full picture of immunological profiles and characteristics in ASD children. Results of this study demonstrate only associations but not causations. Further studies are warranted to reveal the cause–effect relationships among IFN-γ levels, gut microbiota composition and autistic behavioral symptoms.

Despite of these limitations and the preliminary nature of this study, our results suggest that levels of IFN-γ derived from γδT cell could serve as one of the potential candidate biomarkers to subtype ASD individuals to reduce heterogeneity and produce subgroups which are more likely to share a more similar phenotype and etiology. Our results also further support the notion that there exits comprehensive and complex interaction among gut microbiota, immune function and autistic phenotypes. And a better understanding of the associations between immune function and gut microbiota composition as well as metabolism abnormalities in ASD would provide us deep insights into the pathogenesis of ASD and give us important clues to facilitate the development of systemic biomedical treatment for this complex neurodevelopmental disorder.

## Data availability statement

The datasets presented in this study can be found in online repositories. The names of the repository/repositories and accession number(s) can be found below: https://www.ncbi.nlm.nih.gov/, PRJNA530620.

## Ethics statement

The studies involving human participants were reviewed and approved by the Institutional Review Board of Peking Union Medical College Hospital. Written informed consent to participate in this study was provided by the participants’ legal guardian/next of kin.

## Author contributions

X-JX, XY, X-FZ, and GT, conceptualization. X-JX, J-DL, and JY, methodology. J-DL and BL, validation. X-JX, J-DL, JY, and X-DL, formal analysis. X-JX, JY, and XY, investigation. X-JX and JY, writing—original draft preparation. X-JX, J-DL, and XY, writing—review and editing. X-JX and J-DL, visualization. XY and X-FZ, supervision. X-JX and XY, project administration and funding acquisition. All authors contributed to the article and approved the submitted version.

## References

[1] American Psychiatric Association. Diagnostic and statistical manual of mental disorders. 5th ed. Arlington, VA: American Psychiatric Association (2013). doi: 10.1176/appi.books.9780890425596.744053

[2] Hallmayer J Cleveland S Torres A Phillips J Cohen B Torigoe T. Genetic heritability and shared environmental factors among twin pairs with autism. Arch Gen Psychiatry (2011) 68(11):1095–102. doi: 10.1001/archgenpsychiatry.2011.76 PMC4440679 21727249

[3] Sandin S Lichtenstein P Kuja-Halkola R Larsson H Hultman CM Reichenberg A. The familial risk of autism. JAMA (2014) 311(17):1770–7. doi: 10.1001/jama.2014.4144 PMC4381277 24794370

[4] Xu XJ Shou XJ Li J Jia MX Zhang JS Guo Y. Mothers of autistic children: lower plasma levels of oxytocin and arg-vasopressin and a higher level of testosterone. PloS One (2013) 8(9):e74849. doi: 10.1371/journal.pone.0074849 24086383 PMC3783493

[5] Xu XJ Zhang HF Shou XJ Li J Jing WL Zhou Y. Prenatal hyperandrogenic environment induced autistic-like behavior in rat offspring. Physiol Behav (2015) 138:13–20. doi: 10.1016/j.physbeh.2014.09.014 25455866

[6] Wing L Gould J. Severe impairments of social interaction and associated abnormalities in children: Epidemiology and classification. J Autism Dev Disord (1979) 9(1):11–29. doi: 10.1007/BF01531288 155684

[7] O’Brien SK. The validity and reliability of the wing subgroups questionnaire. J Autism Dev Disord (1996) 26(3):321–35. doi: 10.1007/BF02172477 8792263

[8] Rubeis SD He X Goldberg AP Poultney CS Samocha K Cicek AE. Synaptic, transcriptional and chromatin genes disrupted in autism. Nature (2014) 515(7526):209–15. doi: 10.1038/nature13772 PMC4402723 25363760

[9] Meng FC Xu XJ Song TJ Shou XJ Wang XL Han SP. Development of an autism subtyping questionnaire based on social behaviors. Neurosci Bull (2018) 34(5):789–800. doi: 10.1007/s12264-018-0224-8 29633087 PMC6129242

[10] Tager-Flusberg H. Defining language impairments in a subgroup of children with autism spectrum disorder. Sci China Life Sci (2015) 58(10):1044–52. doi: 10.1007/s11427-012-4297-8 26335733

[11] Bernier R Golzio C Xiong B Stessman HA Coe BP Penn O. Disruptive CHD8 mutations define a subtype of autism early in development. Cell (2014) 158(2):263–76. doi: 10.1016/j.cell.2014.06.017 PMC4136921 24998929

[12] McDougle CJ Landino SM Vahabzadeh A O’Rourke J Zurcher NR Finger BC. Toward an immune-mediated subtype of autism spectrum disorder. Brain Res (2015) 1617:72–92. doi: 10.1016/j.brainres.2014.09.048 25445995

[13] Zhang R Jia MX Zhang JS Xu XJ Shou XJ Zhang XT. Transcutaneous electrical acupoint stimulation in children with autism and its impact on plasma levels of arginine-vasopressin and oxytocin: A prospective single-blinded controlled study. Res Dev Disabil (2012) 33(4):1136–46. doi: 10.1016/j.ridd.2012.02.001 22502839

[14] Rapin I Dunn MA Allen DA Stevens MC Fein D. Subtypes of language disorders in school-age children with autism. Dev Neuropsychol (2009) 34(1):66–84. doi: 10.1080/87565640802564648 19142767

[15] Beglinger LJ Smith TH. A review of subtyping in autism and proposed dimensional classification model. J Autism Dev Disord (2001) 31(4):411–22. doi: 10.1023/a:1010616719877 11569587

[16] Volkmar FR Cohen DJ Bregman JD Hooks MY Stevenson JM. An examination of social typologies in autism. J Am Acad Child Adolesc Psychiatry (1989) 28(1):82–6. doi: 10.1097/00004583-198901000-00015 2914840

[17] Wang M Zhou J He F Cai C Wang H Wang Y. Alteration of gut microbiota-associated epitopes in children with autism spectrum disorders. Brain Behav Immun (2019) 75:192–9. doi: 10.1016/j.bbi.2018.10.006 30394313

[18] Tye C Runicles AK Whitehouse AJO Alvares GA. Characterizing the interplay between autism spectrum disorder and comorbid medical conditions: An integrative review. Front Psychiatry (2019) 9:751. doi: 10.3389/fpsyt.2018.00751 30733689 PMC6354568

[19] Yasuhara A. Correlation between EEG abnormalities and symptoms of autism spectrum disorder (ASD). Brain Dev (2010) 32(10):791–8. doi: 10.1016/j.braindev.2010.08.010 20826075

[20] McDougle CJ. Another step toward defining an immune-mediated subtype of autism spectrum disorder. JAMA Netw Open (2018) 1(2):e180280. doi: 10.1001/jamanetworkopen.2018.0280 30646065

[21] Zerbo O Leong A Barcellos L Bernal P Fireman B Croen LA. Immune mediated conditions in autism spectrum disorders. Brain Behav Immun (2015) 46:232–6. doi: 10.1016/j.bbi.2015.02.001 PMC4414798 25681541

[22] Mazefsky CA Schreiber DR Olino TM Minshew NJ. The association between emotional and behavioral problems and gastrointestinal symptoms among children with high-functioning autism. Autism (2014) 18(5):493–501. doi: 10.1177/1362361313485164 24104507 PMC3980202

[23] Kang DW Park JG Ilhan ZE Wallstrom G LaBaer J Adams JB. Reduced incidence of prevotella and other fermenters in intestinal microflora of autistic children. PloS One (2013) 8(7):e68322. doi: 10.1371/journal.pone.0068322 23844187 PMC3700858

[24] Croen LA Zerbo O Qian Y Massolo ML Rich S Sidney S. The health status of adults on the autism spectrum. Autism (2015) 19(7):814–23. doi: 10.1177/1362361315577517 25911091

[25] Jyonouchi H Geng L Davidow AL. Cytokine profiles by peripheral blood monocytes are associated with changes in behavioral symptoms following immune insults in a subset of ASD subjects: An inflammatory subtype? J Neuroinflamm (2014) 11:187. doi: 10.1186/s12974-014-0187-2 PMC4213467 25344730

[26] Gober HJ Kistowska M Angman L Jenö P Mori L Libero GD. Human T cell receptor γδ cells recognize endogenous mevalonate metabolites in tumor cells. J Exp Med (2003) 197(2):163–8. doi: 10.1084/jem.20021500 PMC2193814 12538656

[27] Hu C Qian L Miao Y Huang Q Miao P Wang P. Antigen-presenting effects of effector memory Vγ9VδT cells in rheumatoid arthritis. Cell Mol Immunol (2012) 9(3):245–54. doi: 10.1038/cmi.2011.50 PMC4012843 22139198

[28] Ahmad SF Nadeem A Ansari MA Bakheet SA Al-Ayadhi LY Attia SM. Upregulation of IL-9 and JAK-STAT signaling pathway in children with autism. Prog Neuropsychopharmacol Biol Psychiatry (2017) 79(Pt B):472–80. doi: 10.1016/j.pnpbp.2017.08.002 28802860

[29] Li X Chauhan A Sheikh AM Patil S Chauhan V Li XM. Elevated immune response in the brain of autistic patients. J Neuroimmunol (2009) 207(1-2):111–6. doi: 10.1016/j.jneuroim.2008.12.002 PMC2770268 19157572

[30] Singh VK. Plasma increase of interleukin-12 and interferon-gamma. pathological significance in autism. J Neuroimmunol (1996) 66(1-2):143–5. doi: 10.1016/0165-5728(96)00014-8 8964908

[31] Xu N Li X Zhong Y. Inflammatory cytokines: Potential biomarkers of immunologic dysfunction in autism spectrum disorders. Mediators Inflammation (2015) 2015:531518. doi: 10.1155/2015/531518 PMC4333561 25729218

[32] D’Amelio P Sassi F. Gut microbiota, immune system, and bone. Calcif Tissue Int (2018) 102(4):415–25. doi: 10.1007/s00223-017-0331-y 28965190

[33] Burcelin R. Gut microbiota and immune crosstalk in metabolic disease. Mol Metab (2016) 5(9):771–81. doi: 10.1016/j.molmet.2016.05.016 PMC5004167 27617200

[34] Shi N Li N Duan X Niu H. Interaction between the gut microbiome and mucosal immune system. Mil Med Res (2017) 4:14. doi: 10.1186/s40779-017-0122-9 28465831 PMC5408367

[35] Pickard JM Zeng MY Caruso R Núñez G. Gut microbiota: Role in pathogen colonization, immune responses, and inflammatory disease. Immunol Rev (2017) 279(1):70–89. doi: 10.1111/imr.12567 28856738 PMC5657496

[36] Rose DR Yang H Serena G Sturgeon C Ma B Careaga M. Differential immune responses and microbiota profiles in children with autism spectrum disorders and co-morbid gastrointestinal symptoms. Brain Behav Immun (2018) 70:354–68. doi: 10.1016/j.bbi.2018.03.025 PMC5953830 29571898

[37] Vuong HE Hsiao EY. Emerging roles for the gut microbiome in autism spectrum disorder. Biol Psychiatry (2017) 81(5):411–23. doi: 10.1016/j.biopsych.2016.08.024 PMC5285286 27773355

[38] Li Q Han Y Dy ABC Hagerman RJ. The gut microbiota and autism spectrum disorders. Front Cell Neurosci (2017) 11:120. doi: 10.3389/fncel.2017.00120 28503135 PMC5408485

[39] Coretti L Paparo L Riccio MP Amato F Cuomo M Natale A. Gut microbiota features in young children with autism spectrum disorders. Front Microbiol (2018) 9:3146. doi: 10.3389/fmicb.2018.03146 30619212 PMC6305749

[40] Luna RA Savidge TC Williams KC. The brain-gut-microbiome axis: What role does it play in autism spectrum disorder? Curr Dev Disord Rep (2016) 3(1):75–81. doi: 10.1007/s40474-016-0077-7 27398286 PMC4933016

[41] Kelly JR Minuto C Cryan JF Clarke G Dinan TG. Cross talk: The microbiota and neurodevelopmental disorders. Front Neurosci (2017) 11:490. doi: 10.3389/fnins.2017.00490 28966571 PMC5605633

[42] Ghaisas S Maher J Kanthasamy A. Gut microbiome in health and disease: Linking the microbiome-gut-brain axis and environmental factors in the pathogenesis of systemic and neurodegenerative diseases. Pharmacol Ther (2016) 158:52–62. doi: 10.1016/j.pharmthera.2015.11.012 26627987 PMC4747781

[43] Dinan TG Cryan JF. The microbiome-gut-brain axis in health and disease. Gastroenterol Clin North Am (2017) 46(1):77–89. doi: 10.1016/j.gtc.2016.09.007 28164854

[44] Rellini E Tortolani D Trillo S Carbone S Montecchi F. Childhood autism rating scale (CARS) and autism behavior checklist (ABC) correspondence and conflicts with DSM-IV criteria in diagnosis of autism. J Autism Dev Disord (2004) 34(6):703–8. doi: 10.1007/s10803-004-5290-2 15679189

[45] Geier DA Kern JK Geier MR. A comparison of the autism treatment evaluation checklist (ATEC) and the childhood autism rating scale (CARS) for the quantitative evaluation of autism. J Ment Health Res Intellect Disabil (2013) 6(4):255–67. doi: 10.1080/19315864.2012.681340 PMC3725669 23914277

[46] Duffy FH Shankardass A McAnulty GB Eksioglu YZ Coulter D Rotenberg A. Corticosteroid therapy in regressive autism: A retrospective study of effects on the frequency modulated auditory evoked response (FMAER), language, and behavior. BMC Neurol (2014) 14:70. doi: 10.1186/1471-2377-14-70 24885033 PMC4022403

[47] Segata N Waldron L Ballarini A Narasimhan V Jousson O Huttenhower C. Metagenomic microbial community profiling using unique clade-specific marker genes. Nat Methods (2012) 9(8):811–4. doi: 10.1038/nmeth.2066 PMC3443552 22688413

[48] Segata N Izard J Waldron L Gevers D Miropolsky L Garrett WS. Metagenomic biomarker discovery and explanation. Genome Biol (2011) 12(6):R60. doi: 10.1186/gb-2011-12-6-r60 21702898 PMC3218848

[49] Li R Yu C Li Y Lam TW Yiu SM Kristiansen K. SOAP2: An improved ultrafast tool for short read alignment. Bioinformatics (2009) 25(15):1966–7. doi: 10.1093/bioinformatics/btp336 19497933

[50] Li J Jia H Cai X Zhong H Feng Q Sunagawa S. An integrated catalog of reference genes in the human gut microbiome. Nat Biotechnol (2014) 32(8):834–41. doi: 10.1038/nbt.2942 24997786

[51] Drula E Garron ML Dogan S Lombard V Henrissat B Terrapon N. The carbohydrate-active enzyme database: functions and literature. Nucleic Acids Res (2022) 50(D1):D571–7. doi: 10.1093/nar/gkab1045 PMC8728194 34850161

[52] Urban M Cuzick A Seager J Wood V Rutherford K Venkatesh SY. PHI-base: the pathogen-host interactions database. Nucleic Acids Res (2020) 48(D1):D613–20. doi: 10.1093/nar/gkz904 PMC7145647 31733065

[53] Valles-Colomer M Falony G Darzi Y Tigchelaar EF Wang J Tito RY. The neuroactive potential of the human gut microbiota in quality of life and depression. Nat Microbiol (2019) 4(4):623–32. doi: 10.1038/s41564-018-0337-x 30718848

[54] Chong J Wishart DS Xia J. Using MetaboAnalyst 4.0 for comprehensive and integrative metabolomics data analysis. Curr Protoc Bioinf (2019) 68(1):e86. doi: 10.1002/cpbi.86 31756036

[55] Masi A DeMayo MM Glozier N Guastella AJ. An overview of autism spectrum disorder, heterogeneity and treatment options. Neurosci Bull (2017) 33(2):183–93. doi: 10.1007/s12264-017-0100-y PMC5360849 28213805

[56] Tordjman S Cohen D Coulon N Anderson GM Botbol M Canitano R. Reframing autism as a behavioral syndrome and not a specific mental disorder: Implications of genetic and phenotypic heterogeneity. Neurosci Biobehav Rev (2017) 80:210. doi: 10.1016/j.neubiorev.2017.01.030 28153685

[57] An JY Claudianos C. Genetic heterogeneity in autism: From single gene to a pathway perspective. Neurosci Biobehav Rev (2016) 68:442–53. doi: 10.1016/j.neubiorev.2016.06.013 27317861

[58] Newschaffer CJ Fallin D Lee NL. Heritable and nonheritable risk factors for autism spectrum disorders. Epidemiol Rev (2002) 24(2):137–53. doi: 10.1093/epirev/mxf010 12762089

[59] Hong RP Hou YY Xu XJ Lang JD Jin YF Zeng XF. The difference of gut microbiota and their correlations with urinary organic acids between autistic children with and without atopic dermatitis. Front Cell Infect Microbiol (2022) 12:886196. doi: 10.3389/fcimb.2022.886196 35800387 PMC9253573

[60] Warren RP Margaretten NC Pace NC Foster A. Immune abnormalities in patients with autism. J Autism Dev Disord (1986) 16(2):189–97. doi: 10.1007/BF01531729 2941410

[61] Wu S Ding Y Wu F Li R Xie G Hou J. Family history of autoimmune diseases is associated with an increased risk of autism in children: A systematic review and meta-analysis. Neurosci Biobehav Rev (2015) 55:322–32. doi: 10.1016/j.neubiorev.2015.05.004 25981892

[62] Garay PA Hsiao EY Patterson PH McAllister AK. Maternal immune activation causes age- and region-specific changes in brain cytokines in offspring throughout development. Brain Behav Immun (2013) 31:54–68. doi: 10.1016/j.bbi.2012.07.008 22841693 PMC3529133

[63] Schneider CK Melmed RD Barstow LE Enriquez FJ Ranger-Moore J Ostrem JA. Oral human immunoglobulin for children with autism and gastrointestinal dysfunction: A prospective, open-label study. J Autism Dev Disord (2006) 36(8):1053–64. doi: 10.1007/s10803-006-0141-y 16845577

[64] Asadabadi M Mohammadi MR Ghanizadeh A Modabbernia A Ashrafi M Hassanzadeh E. Celecoxib as adjunctive treatment to risperidone in children with autistic disorder: A randomized, double-blind, placebo-controlled trial. Psychopharmacol (Berl) (2013) 225(1):51–9. doi: 10.1007/s00213-012-2796-8 22782459

[65] Akhondzadeh S Fallah J Mohammadi MR Imani R Mohammadi M Salehi B. Double-blind placebo-controlled trial of pentoxifylline added to risperidone: Effects on aberrant behavior in children with autism. Prog Neuropsychopharmacol Biol Psychiatry (2010) 34(1):32–6. doi: 10.1016/j.pnpbp.2009.09.012 19772883

[66] Boris M Kaiser CC Goldblatt A Elice MW Edelson SM Adams JB. Effect of pioglitazone treatment on behavioral symptoms in autistic children. J Neuroinflamm (2007) 4:3. doi: 10.1186/1742-2094-4-3 PMC1781426 17207275

[67] Melandri D Zlatareva I Chaleil RAG Dart RJ Chancellor A Nussbaumer O. The γδTCR combines innate immunity with adaptive immunity by utilizing spatially distinct regions for agonist selection and antigen responsiveness. Nat Immunol (2018) 19(12):1352–65. doi: 10.1038/s41590-018-0253-5 PMC6874498 30420626

[68] Alfawaz HA Bhat RS Al-Ayadhi L El-Ansary AK. Protective and restorative potency of vitamin d on persistent biochemical autistic features induced in propionic acid-intoxicated rat pups. BMC Complement Altern Med (2014) 14:416. doi: 10.1186/1472-6882-14-416. D2. 25344727 PMC4230722

[69] Zhang Y Gao D Kluetzman K Mendoza A Bolivar VJ Reilly A. The maternal autoimmune environment affects the social behavior of offspring. J Neuroimmunol (2013) 258(1-2):51–60. doi: 10.1016/j.jneuroim.2013.02.019 23537887

[70] Sweeten TL Posey DJ Shankar S McDougle CJ. High nitric oxide production in autistic disorder: a possible role for interferon-gamma. Biol Psychiatry (2004) 55(4):434–7. doi: 10.1016/j.biopsych.2003.09.001 14960298

[71] Jyonouchi H Sun S Itokazu N. Innate immunity associated with inflammatory responses and cytokine production against common dietary proteins in patients with autism spectrum disorder. Neuropsychobiology (2002) 46(2):76–84. doi: 10.1159/000065416 12378124

[72] Patel N Crider A Pandya CD Ahmed AO Pillai A. Altered mRNA levels of glucocorticoid receptor, mineralocorticoid receptor, and Co-chaperones (FKBP5 and PTGES3) in the middle frontal gyrus of autism spectrum disorder subjects. Mol Neurobiol (2016) 53(4):2090–9. doi: 10.1007/s12035-015-9178-2 25912394

[73] Sapolsky RM. The physiological relevance of glucocorticoid endangerment of the hippocampus. Ann N Y Acad Sci (1994) 746:294–307. doi: 10.1111/j.1749-6632.1994.tb39247.x 7825884

[74] Saghazadeh A Ataeinia B Keynejad K Abdolalizadeh A Hirbod-Mobarakeh A Rezaei N. A meta-analysis of pro-inflammatory cytokines in autism spectrum disorders: Effects of age, gender, and latitude. J Psychiatr Res (2019) 115:90–102. doi: 10.1016/j.jpsychires.2019.05.019 31125917

[75] Nour-Eldine W Ltaief SM Abdul Manaph NP Al-Shammari AR. In search of immune cellular sources of abnormal cytokines in the blood in autism spectrum disorder: A systematic review of case-control studies. Front Immunol (2022) 13:950275. doi: 10.3389/fimmu.2022.950275 36268027 PMC9578337

[76] Finegold SM Dowd SE Gontcharova V Liu C Henley KE Wolcott RD. Pyrosequencing study of fecal microflora of autistic and control children. Anaerobe (2010) 16(4):444–53. doi: 10.1016/j.anaerobe.2010.06.008 20603222

[77] Strati F Cavalieri D Albanese D De Felice C Donati C Hayek J. New evidences on the altered gut microbiota in autism spectrum disorders. Microbiome (2017) 5(1):24. doi: 10.1186/s40168-017-0242-1 28222761 PMC5320696

[78] Sutcliffe IC. A phylum level perspective on bacterial cell envelope architecture. Trends Microbiol (2010) 18(10):464–70. doi: 10.1016/j.tim.2010.06.005 20637628

[79] Tomova A Husarova V Lakatosova S Bakos J Vlkova B Babinska K. Gastrointestinal microbiota in children with autism in Slovakia. Physiol Behav (2015) 138:179–87. doi: 10.1016/j.physbeh.2014.10.033 25446201

[80] Koliada K Syzenko G Moseiko V Budovska L Puchkov K Perederiy V. Association between body mass index and Firmicutes/Bacteroidetes ratio in an adult Ukrainian population. BMC Microbiol (2017) 17(1):120. doi: 10.1186/s12866-017-1027-1 28532414 PMC5440985

[81] Riva A Borgo F Lassandro C Verduci E Morace G Borghi E. Pediatric obesity is associated with an altered gut microbiota and discordant shifts in firmicutes populations. Environ Microbiol (2017) 19(1):95–105. doi: 10.1111/1462-2920.13463 27450202 PMC5516186

[82] Collado MC Derrien M Isolauri E de Vos WM Salminen S. Intestinal integrity and akkermansia muciniphila, a mucin-degrading member of the intestinal microbiota present in infants, adults, and the elderly. Appl Environ Microbiol (2007) 73(23):7767–70. doi: 10.1128/AEM.01477-07 PMC2168041 17933936

[83] Wang L Christophersen CT Sorich MJ Gerber JP Angley MT Conlon MA. Low relative abundances of the mucolytic bacterium akkermansia muciniphila and bifidobacterium spp. in feces of children with autism. Appl Environ Microbiol (2011) 77(18):6718–21. doi: 10.1128/AEM.05212-11 PMC3187122 21784919

[84] Inoue R Sakaue Y Sawai C Sawai T Ozeki M Romero-Pérez GA. A preliminary investigation on the relationship between gut microbiota and gene expressions in peripheral mononuclear cells of infants with autism spectrum disorders. Biosci Biotechnol Biochem (2016) 80(12):2450–8. doi: 10.1080/09168451.2016.1222267 27581276

[85] Downes J Vartoukian SR Dewhirst FE Izard J Chen T Yu WH. Pyramidobacter piscolens gen. nov., sp. nov., a member of the phylum 'Synergistetes' isolated from the human oral cavity. Int J Syst Evol Microbiol (2009) 59(Pt 5):972–80. doi: 10.1099/ijs.0.000364-0 PMC2868594 19406777

[86] Deng ZL Szafrański SP Jarek M Bhuju S Wagner-Döbler I. Dysbiosis in chronic periodontitis: Key microbial players and interactions with the human host. Sci Rep (2017) 7(1):3703. doi: 10.1038/s41598-017-03804-8 28623321 PMC5473847

[87] Ferrazzano GF Salerno C Bravaccio C Ingenito A Sangianantoni G Cantile T. Autism spectrum disorders and oral health status: review of the literature. Eur J Paediatr Dent (2020) 21(1):9–12. doi: 10.23804/ejpd.2020.21.01.02 32183521

[88] Marchandin H Damay A Roudière L Teyssier C Zorgniotti I Dechaud H. Phylogeny, diversity and host specialization in the phylum synergistetes with emphasis on strains and clones of human origin. Res Microbiol (2010) 161(2):91–100. doi: 10.1016/j.resmic.2009.12.008 20079831

[89] Xie C Teng J Wang X Xu B Niu Y Ma L. Multi-omics analysis reveals gut microbiota-induced intramuscular fat deposition *via* regulating expression of lipogenesis-associated genes. Anim Nutr (2021) 9:84–99. doi: 10.1016/j.aninu.2021.10.010 35949981 PMC9344316

[90] Li G Yin P Chu S Gao W Cui S Guo S. Correlation analysis between GDM and gut microbial composition in late pregnancy. J Diabetes Res (2021) 2021:8892849. doi: 10.1155/2021/8892849 33628840 PMC7889370

[91] Lawson PA Song Y Liu C Molitoris DR Vaisanen ML Collins MD. Anaerotruncus colihominis gen. nov., sp. nov., from human faeces. Int J Syst Evol Microbiol (2004) 54(Pt 2):413–7. doi: 10.1099/ijs.0.02653-0 15023953

[92] Yao Y Yan L Chen H Wu N Wang W Wang D. Cyclocarya paliurus polysaccharides alleviate type 2 diabetic symptoms by modulating gut microbiota and short-chain fatty acids. Phytomedicine (2020) 77:153268. doi: 10.1016/j.phymed.2020.153268 32663709

[93] Satokari R Fuentes S Mattila E Jalanka J de Vos WM Arkkila P. Fecal transplantation treatment of antibiotic-induced, noninfectious colitis and long-term microbiota follow-up. Case Rep Med (2014) 2014:913867. doi: 10.1155/2014/913867 25548572 PMC4274837

[94] Lee JY Mannaa M Kim Y Kim J Kim GT Seo YS. Comparative analysis of fecal microbiota composition between rheumatoid arthritis and osteoarthritis patients. Genes (Basel) (2019) 10(10):748. doi: 10.3390/genes10100748 31557878 PMC6827100

[95] Jeong S Huang LK Tsai MJ Liao YT Lin YS Hu CJ. Cognitive function associated with gut microbial abundance in sucrose and s-Adenosyl-L-Methionine (SAMe) metabolic pathways. J Alzheimers Dis (2022) 87(3):1115–30. doi: 10.3233/JAD-215090 35431236

[96] Kato M. New insights into IFN-γ in rheumatoid arthritis: role in the era of JAK inhibitors. Immunol Med (2020) 43(2):72–8. doi: 10.1080/25785826.2020.1751908 32338187

[97] Wang L Christophersen CT Sorich MJ Gerber JP Angley MT Conlon MA. Elevated fecal short chain fatty acid and ammonia concentrations in children with autism spectrum disorder. Dig Dis Sci (2012) 57(8):2096–102. doi: 10.1007/s10620-012-2167-7 22535281

[98] Kau AL Ahern PP Griffin NW Goodman AL Gordon JI. Human nutrition, the gut microbiome and the immune system. Nature (2011) 474(7351):327–36. doi: 10.1038/nature10213 PMC3298082 21677749

[99] Ulsemer P Toutounian K Kressel G Schmidt J Karsten U Hahn A. Safety and tolerance of bacteroides xylanisolvens DSM 23964 in healthy adults. Benef Microbes (2012) 3(2):99–111. doi: 10.3920/BM2011.0051 22417778

[100] de Vrese M Winkler P Rautenberg P Harder T Noah C Laue C. Effect of lactobacillus gasseri PA 16/8, bifidobacterium longum SP 07/3, b. bifidum MF 20/5 on common cold episodes: A double blind, randomized, controlled trial. Clin Nutr (2005) 24(4):481–91. doi: 10.1016/j.clnu.2005.02.006 16054520

[101] Wang H Lee IS Braun C Enck P. Effect of probiotics on central nervous system functions in animals and humans: A systematic review. J Neurogastroenterol Motil (2016) 22(4):589–605. doi: 10.5056/jnm16018 27413138 PMC5056568

[102] Mirande C Kadlecikova E Matulova M Capek P Bernalier-Donadille A Forano E. Dietary fibre degradation and fermentation by two xylanolytic bacteria bacteroides xylanisolvens XB1AT and roseburia intestinalis XB6B4 from the human intestine. J Appl Microbiol (2010) 109(2):451–60. doi: 10.1111/j.1365-2672.2010.04671.x 20105245

[103] Chassard C Delmas E Lawson PA Bernalier-Donadille A. Bacteroides xylanisolvens sp. nov., a xylandegrading bacterium isolated from human faeces. Int J Syst Evol Microbiol (2008) 58(Pt 4):1008–13. doi: 10.1099/ijs.0.65504-0 18398210

[104] Thaiss CA Levy M Suez J Elinav E. The interplay between the innate immune system and the microbiota. Curr Opin Immunol (2014) 26:41–8. doi: 10.1016/j.coi.2013.10.016 24556399

[105] Hsiao EY McBride SW Hsien S Sharon G Hyde ER McCue T. Microbiota modulate behavioral and physiological abnormalities associated with neurodevelopmental disorders. Cell (2013) 155(7):1451–63. doi: 10.1016/j.cell.2013.11.024 PMC3897394 24315484

[106] Gilbert JA Krajmalnik-Brown R Porazinska DL Weiss SJ Knight R. Toward effective probiotics for autism and other neurodevelopmental disorders. Cell (2013) 155(7):1446–8. doi: 10.1016/j.cell.2013.11.035 PMC4166551 24360269

[107] Schell MA Karmirantzou M Snel B Vilanova D Berger B Pessi G. The genome sequence of bifidobacterium longum reflects its adaptation to the human gastrointestinal tract. Proc Natl Acad Sci U.S.A. (2002) 99(22):14422–7. doi: 10.1073/pnas.212527599 PMC137899 12381787

[108] Lievin V Peiffer I Hudault S Rochat F Brassart D Neeser JR. Bifidobacterium strains from resident infant human gastrointestinal microflora exert antimicrobial activity. Gut (2000) 47(5):646–52. doi: 10.1136/gut.47.5.646 PMC1728100 11034580

[109] Tanaka H Hashiba H Kok J Mierau I. Bile salt hydrolase of bifidobacterium longum - biochemical and genetic characterization. Appl Environ Microbiol (2000) 66(6):2502–12. doi: 10.1128/AEM.66.6.2502-2512.2000 PMC110569 10831430

[110] Sela DA Chapman J Adeuya A Kim JH Chen F Whitehead TR. The genome sequence of bifidobacterium longum subsp. infantis reveals adaptations for milk utilization within the infant microbiome. Proc Natl Acad Sci U.S.A. (2008) 105(48):18964–9. doi: 10.1073/pnas.0809584105 PMC2596198 19033196

[111] Ma X Shin YJ Jang HM Joo MK Yoo JW Kim DH. Lactobacillus rhamnosus and bifidobacterium longum alleviate colitis and cognitive impairment in mice by regulating IFN-γ to IL-10 and TNF-α to IL-10 expression ratios. Sci Rep (2021) 11(1):20659. doi: 10.1038/s41598-021-00096-x 34667205 PMC8526673

[112] De Angelis M Piccolo M Vannini L Siragusa S De Giacomo A Serrazzanetti DI. Fecal microbiota and metabolome of children with autism and pervasive developmental disorder not otherwise specified. PloS One (2013) 8(10):e76993. doi: 10.1371/journal.pone.0076993 24130822 PMC3793965

[113] Adams JB Johansen LJ Powell LD Quig D Rubin RA. Gastrointestinal flora and gastrointestinal status in children with autism - comparisons to typical children and correlation with autism severity. BMC Gastroenterol (2011) 11:22. doi: 10.1186/1471-230X-11-22 21410934 PMC3072352

[114] Cani PD. Interactions between gut microbes and host cells control gut barrier and metabolism. Int J Obes Suppl (2016) 6(Suppl 1):S28–31. doi: 10.1038/ijosup.2016.6 PMC5485881 28685027

[115] Mussap M Noto A Fanos V. Metabolomics of autism spectrum disorders: early insights regarding mammalian-microbial cometabolites. Expert Rev Mol Diagn (2016) 16(8):869–81. doi: 10.1080/14737159.2016.1202765 27310602

[116] Bugajska J Berska J Wojtyto T Bik-Multanowski M Sztefko K. The amino acid profile in blood plasma of young boys with autism. Psychiatr Pol (2017) 51(2):359–68. doi: 10.12740/PP/65046 28581543

[117] Bala KA Doğan M Mutluer T Kaba S Aslan O Balahoroğlu R. Plasma amino acid profile in autism spectrum disorder (ASD). Eur Rev Med Pharmacol Sci (2016) 20(5):923–9. Available at: https://www.europeanreview.org/wp/wp-content/uploads/923-929.pd. 27010152

[118] Xu XJ Cai XE Meng FC Song TJ Wang XX Wei YZ. Comparison of the metabolic profiles in the plasma and urine samples between autistic and typically developing boys: a preliminary study. Front Psychiatry (2021) 12:657105. doi: 10.3389/fpsyt.2021.657105 34149478 PMC8211775

[119] Diémé B Mavel S Blasco H Tripi G Bonnet-Brilhault F Malvy J. Metabolomics study of urine in autism spectrum disorders using a multiplatform analytical methodology. J Proteome Res (2015) 14(12):5273–82. doi: 10.1021/acs.jproteome.5b00699 26538324

[120] Clayton TA. Metabolic differences underlying two distinct rat urinary phenotypes, a suggested role for gut microbial metabolism of phenylalanine and a possible connection to autism. FEBS Lett (2012) 586(7):956–61. doi: 10.1016/j.febslet.2012.01.049 22306194

[121] Williams BL Hornig M Buie T Bauman ML Cho Paik M Wick I. Impaired carbohydrate digestion and transport and mucosal dysbiosis in the intestines of children with autism and gastrointestinal disturbances. PloS One (2011) 6(9):e24585. doi: 10.1371/journal.pone.0024585 21949732 PMC3174969

[122] Brigandi SA Shao H Qian SY Shen Y Wu BL Kang JX. Autistic children exhibit decreased levels of essential fatty acids in red blood cells. Int J Mol Sci (2015) 16(5):10061–76. doi: 10.3390/ijms160510061 PMC4463632 25946342

[123] Kushak RI Winter HS Buie TM Cox SB Phillips CD Ward NL. Analysis of the duodenal microbiome in autistic individuals: association with carbohydrate digestion. J Pediatr Gastroenterol Nutr (2017) 64(5):e110–6. doi: 10.1097/MPG.0000000000001458 27811623

[124] Fernandez-Julia PJ Munoz-Munoz J van Sinderen D. A comprehensive review on the impact of β-glucan metabolism by bacteroides and bifidobacterium species as members of the gut microbiota. Int J Biol Macromol (2021) 181:877–89. doi: 10.1016/j.ijbiomac.2021.04.069 33864864

[125] Wong JM de Souza R Kendall CW Emam A Jenkins DJ. Colonic health: Fermentation and short chain fatty acids. J Clin Gastroenterol (2006) 40(3):235–43. doi: 10.1097/00004836-200603000-00015 16633129

[126] Vijay N Morris ME. Role of monocarboxylate transporters in drug delivery to the brain. Curr Pharm Des (2014) 20(10):1487–98. doi: 10.2174/13816128113199990462 PMC4084603 23789956

[127] Morris G Berk M Carvalho A Caso JR Sanz Y Walder K. The role of the microbial metabolites including tryptophan catabolites and short chain fatty acids in the pathophysiology of immune-inflammatory and neuroimmune disease. Mol Neurobiol (2017) 54(6):4432–51. doi: 10.1007/s12035-016-0004-2 27349436

[128] MacFabe DF. Short-chain fatty acid fermentation products of the gut microbiome: implications in autism spectrum disorders. Microb Ecol Health Dis (2012) 23:19260. doi: 10.3402/mehd.v23i0.19260 PMC3747729 23990817

[129] Innis SM. The role of dietary n-6 and n-3 fatty acids in the developing brain. Dev Neurosci (2000) 22(5-6):474–80. doi: 10.1159/000017478 11111165

[130] Richardson AJ. Long-chain polyunsaturated fatty acids in childhood developmental and psychiatric disorders. Lipids (2004) 39(12):1215–22. doi: 10.1007/s11745-004-1350-z 15736918

[131] Madra M Ringel R Margolis KG. Gastrointestinal issues and autism spectrum disorder. Child Adolesc Psychiatr Clin N Am (2020) 29(3):501–13. doi: 10.1016/j.chc.2020.02.005 PMC8608248 32471598

[132] Wright C Shin JH Rajpurohit A Deep-Soboslay A Collado-Torres L Brandon NJ. Altered expression of histamine signaling genes in autism spectrum disorder. Transl Psychiatry (2017) 7(5):e1126. doi: 10.1038/tp.2017.87 28485729 PMC5534955

[133] Linday LA Tsiouris JA Cohen IL Shindledecker R DeCresce R. Famotidine treatment of children with autistic spectrum disorders: pilot research using single subject research design. J Neural Transm (Vienna) (2001) 108(5):593–611. doi: 10.1007/s007020170059 11459079

[134] Molenhuis RT Hutten L Kas MJH. Histamine H3 receptor antagonism modulates autism-like hyperactivity but not repetitive behaviors in BTBR T+Itpr3tf/J inbred mice. Pharmacol Biochem Behav (2022) 212:173304. doi: 10.1016/j.pbb.2021.173304 34856309

[135] Eissa N Azimullah S Jayaprakash P Jayaraj RL Reiner D Ojha SK. The dual-active histamine H3 receptor antagonist and acetylcholine esterase inhibitor E100 alleviates autistic-like behaviors and oxidative stress in valproic acid induced autism in mice. Int J Mol Sci (2020) 21(11):3996. doi: 10.3390/ijms21113996 32503208 PMC7312782

[136] Kamei M Otani Y Hayashi H Nakamura T Yanai K Furuta K. Suppression of IFN-γ production in murine splenocytes by histamine receptor antagonists. Int J Mol Sci (2018) 19(12):4083. doi: 10.3390/ijms19124083 30562962 PMC6321562

[137] Kohda F Koga T Uchi H Urabe K Furue M. Histamine-induced IL-6 and IL-8 production are differentially modulated by IFN-gamma and IL-4 in human keratinocytes. J Dermatol Sci (2002) 28(1):34–41. doi: 10.1016/s0923-1811(01)00147-5 11916128

[138] Khalaj M Saghazadeh A Shirazi E Shalbafan MR Alavi K Shooshtari MH. Palmitoylethanolamide as adjunctive therapy for autism: Efficacy and safety results from a randomized controlled trial. J Psychiatr Res (2018) 103:104–11. doi: 10.1016/j.jpsychires.2018.04.022 29807317

[139] Taliou A Zintzaras E Lykouras L Francis K. An open-label pilot study of a formulation containing the anti-inflammatory flavonoid luteolin and its effects on behavior in children with autism spectrum disorders. Clin Ther (2013) 35(5):592–602. doi: 10.1016/j.clinthera.2013.04.006 23688534

