# Supplementary material for: Differences of gut microbiota and behavioral symptoms between two subgroups of autistic children based on γδT cells-derived IFN-γ Levels: A preliminary study
Source: Front Immunol. 2023 Feb 15;14:1100816. doi: 10.3389/fimmu.2023.1100816 (PMC9975759; doi:10.3389/fimmu.2023.1100816)
Supplement: Supplementary file 1 [file DataSheet_1.docx]

Supplementary Material

## Supplementary Figures


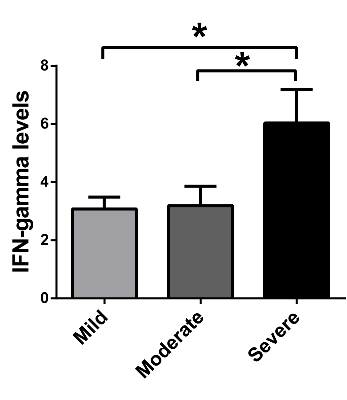


**Supplementary Figure 1. Preliminary analysis of IFN-γ levels vs ASD severity indicated in recent clinical records (graded as mild, moderate or severe).** Data are presented as mean ±SEM. **p*<0.05.


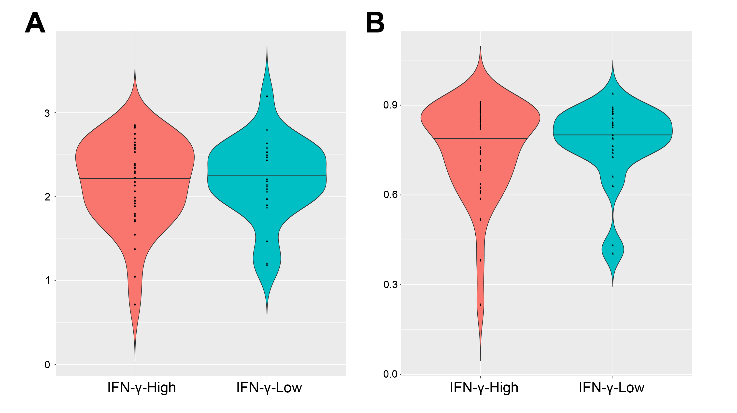


**Supplementary Figure 2.** Comparation of indexes of alpha-diversity of the fecal microbiota between the two groups. (**A**) The Shannon diversity index. (**B**) The Simpson diversity index.


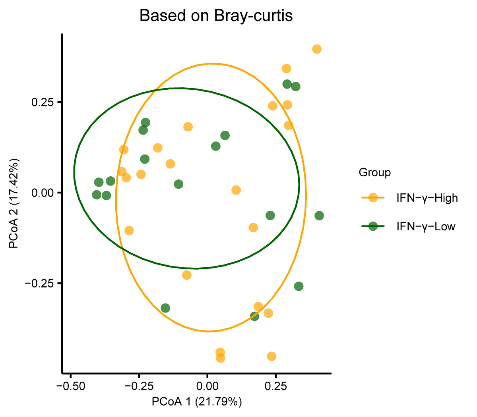


**Supplementary Figure 3.** The principal coordination analysis (PCoA) of bacterial beta diversity of microbiomes based on the Bray–Curtis dissimilarity for ASD children in the IFN-γ-Low and IFN-γ-High groups. (PERMANOVA, *r*^2^= 0.0276, *p*= 0.398).
